# Integrated internal ion-gated organic electrochemical transistors for stand-alone conformable bioelectronics

**DOI:** 10.1038/s41563-023-01599-w

**Published:** 2023-07-10

**Authors:** Claudia Cea, Zifang Zhao, Duncan J. Wisniewski, George D. Spyropoulos, Anastasios Polyravas, Jennifer N. Gelinas, Dion Khodagholy

**Affiliations:** 1 Department of Electrical Engineering, Columbia University, New York, NY, USA; 2 Department of Neurology, Columbia University Medical Center, New York, NY, USA; 3 Institute for Genomic Medicine, Columbia University Medical Center, New York, NY, USA; 4 Present address: Department Information Technology, Waves, UGhent, Technology Campus, iGhent, Zwijnaarde, Belgium; 5 These authors contributed equally: Claudia Cea, Zifang Zhao

## Abstract

Organic electronics can be biocompatible and conformable, enhancing the ability to interface with tissue. However, the limitations of speed and integration have, thus far, necessitated reliance on silicon-based technologies for advanced processing, data transmission and device powering. Here we create a stand-alone, conformable, fully organic bioelectronic device capable of realizing these functions. This device, vertical internal ion-gated organic electrochemical transistor (vIGT), is based on a transistor architecture that incorporates a vertical channel and a miniaturized hydration access conduit to enable megahertz-signal-range operation within densely packed integrated arrays in the absence of crosstalk. These transistors demonstrated long-term stability in physiologic media, and were used to generate high-performance integrated circuits. We leveraged the high-speed and low-voltage operation of vertical internal ion-gated organic electrochemical transistors to develop alternating-current-powered conformable circuitry to acquire and wirelessly communicate signals. The resultant stand-alone device was implanted in freely moving rodents to acquire, process and transmit neurophysiologic brain signals. Such fully organic devices have the potential to expand the utility and accessibility of bioelectronics to a wide range of clinical and societal applications.

It is increasingly appreciated that individual variability can strongly affect response to clinical treatments, motivating approaches that enable the long-term monitoring of physiologic signals and delivery of responsive therapeutics^1,2^. Implanted bioelectronic devices are often critical components of such approaches, but implementation challenges hinder widespread use. The incompatibility of traditional electronic components with physiologic media, risk of device-related tissue disruption and limited means by which to establish an interface with a fully implanted device inside the body for exchange of data and power are key hurdles^3,4^.

Organic semiconductors can be used to form transistor channels and establish inherently flexible devices. Such devices can take the form of organic field-effect transistors (OFETs), barrier-controlled devices wherein the gate potential across the barrier layer can modulate the channel current^5^. Some of these organic semiconductor channel materials are stable in aqueous environments, eliminating the need for an electronic insulator between the gate and channel, resulting in devices known as electrolyte-gated OFETs^6^. In this case, the potential established across the electric double-layer capacitance between the electrolyte and channel controls the channel current^7–9^. This architecture enables the formation of an electric double-layer capacitor, increasing the capacitance, and allowing the same channel material to be operated at substantially lower voltages, which is potentially advantageous for in vivo applications^6^. However, only the surface of the channel contributes to the capacitance, leading to transconductance values typically insufficient to resolve low-amplitude physiologic signals.

To further enhance this capacitance, organic electrochemical transistors use hydrophilic and ion-permeable polymers as the channel material (such as poly(2,3-dihydrothieno-1,4-dioxin)-poly(styrenesu lfonate) (PEDOT:PSS)). These polymers can undergo bulk redox reactions, resulting in volumetric capacitance and high transconductance^10–13^. However, this improvement comes at the cost of longer device time constants, because the temporal dynamics are defined by ion mobility, and the inability to perform independent gating, due to the requisite function of external electrolyte for transistor operation. We previously developed internal ion-gated organic electrochemical transistors (IGTs) to address these limitations of electrolyte-gated OFETs and organic electrochemical transistors^14^. The conducting polymer-based channel of an IGT contains ion reservoirs that are sufficient for effective de/doping of the channel, eliminating the dependence on an external electrolyte and reducing the transit time of ions. These features extend the bandwidth up to several hundreds of kilohertz and permit independent gating to form functional circuits^14–16^. Yet, Si-based transistors operate at higher speeds, particularly for the purposes of operations such as data communication. In addition, when immersed in an ion-conducting medium, such as physiologic tissue, the operation of an IGT can induce a potential in the medium that modulates the gate of a neighbouring IGT and results in crosstalk.

At a systems level, stand-alone bioelectronic devices require components for signal acquisition, processing, data transmission and powering. Traditionally, an implanted device with these capabilities would require multiple rigid and non-biocompatible components, including complex radiofrequency circuitry for communication, batteries for power and a series of interfacing boards to establish physical and electrical connections between these modules^17,18^. Transiently powered and wireless bioelectronics eliminate the need for bulky batteries and cables by harvesting an externally propagated energy source such as radiofrequency, ultrasound and magnetic or ionic waves^19,20^. However, these approaches still require implanted, rigid and encapsulated electronics to convert this energy into a usable form for signal acquisition, processing and transmission modules, precluding the realization of fully conformable stand-alone bioelectronics. Thus, there is an unmet need for circuit-building blocks that concurrently possess all these features: (1) low voltage required for operation to avoid hydrolysis within biologic tissue; (2) biocompatibility, stability and conformability for in vivo operation; and (3) high electrical performance (including fast temporal response, high transconductance and crosstalk-free operation).

Here we directly address these issues by introducing a scalable, self-contained, sub-micrometre IGT architecture, namely, the vIGT. We incorporate a vertical channel arrangement that augments the intrinsic speed of the IGT architecture by optimizing the channel geometry and permits a high-density integration capacity. We also deployed a vertical hydration access conduit (H-via) that maintains device hydration but prevents crosstalk even when immersed in an ion-rich medium. The combination of these features resulted in an organic transistor that is able to operate crosstalk free in the megahertz signal range. Also, vIGTs were stable in physiologic conditions for over one year without the need for encapsulation, and were effectively fabricated in conformable arrays with a density of 155,000 transistors cm^−2^. The reduction state of the channel was chemically tuned to permit device operation in both enhancement and depletion modes. The properties of vIGT permit the development of conformable, wireless circuitry capable not only of acquiring and transmitting physiologic signals but simultaneously providing power to the implanted device. We demonstrated this capacity by performing high-spatiotemporal-resolution electrophysiology in vivo in a rodent model using only fully conformable implanted vIGT-based circuitry in the absence of any rigid or Si-based components. Therefore, vIGTs represent a convergence of high electronic performance, scalability, stability and conformability, capable of serving as the foundation for stand-alone organic bioelectronic devices.

We hypothesized that incorporating the operational principles of IGTs with two material and design elements would generate high-performance, high-speed transistors that could form large-scale integrated circuits with minimal crosstalk, even when fully implanted in physiologic media: (1) vertical channel orientation; (2) introduction of an H-via that attenuates the ionic interaction of the gate with any external medium (Fig. 1a).

To test the feasibility of these concepts, we first fabricated vIGTs guided by processes developed for horizontal IGTs. The composition of vIGTs was similar to horizontal IGTs: a PEDOT:PSS-based channel containing sugar alcohol (d-sorbitol) to create a depletion-mode (normally ON) transistor, with the addition of poly-ethylamine (PEI) to generate an enhancement-mode (normally OFF) transistor^15,21,22^. These additives are highly biocompatible and preserved the electrical properties of the PEDOT:PSS (refs. 23–26). We exploited the facile solution processability of these channel material dispersions to vertically orient the channel. The length of this vertical channel was defined by the thickness of the interlayers (800 nm). The gate electrodes were composed of PEDOT:PSS to increase the charge capacity compared with Au (refs. 27,28). We maintained a polysaccharide-based (chitosan) ion membrane to establish ionic–but prevent electronic–conduction between the gate and channel. To allow for the hydration of the transistor channel, we etched a vertical H-via across the ion membrane layer (Fig. 1b,c). The entire fabrication process was implemented at the wafer scale and used for the creation of both depletion- and enhancement-mode transistors (Supplementary Fig. 1).

We first characterized the steady-state characteristics of vIGTs. They possessed high transconductance and ON current similar to their horizontal IGT counterparts, establishing their capacity for efficient miniaturization. Temporal responses were in the sub-microsecond domain (Fig. 1d). This high-speed operation was achieved for both depletion- and enhancement-mode vIGTs (Supplementary Fig. 2). We found that the overall performance of vIGTs characterized by their transconductance versus rise time ratio (normalized by the channel width) outperformed organic and inorganic flexible transistors such as organic field-effect transistors, electrolyte-gated organic or inorganic field-effect transistors, indium gallium zinc oxide and Si-based foundry-produced transistors (Fig. 1e and Supplementary Table 1).

Next, we investigated the operation of vIGTs within a physiologic environment containing fluids with high ionic conductivity. In dry conditions, patterned electrolytes or anisotropic ion-conducting composites prevent crosstalk between adjacent transistors^29,30^. Here we developed a photolithographic process to pattern chitosan at <5 μm resolution to allow the creation of densely packed vIGTs. In aqueous and ion-conducting environments, these patterning approaches would be insufficient to eliminate coupling between the transistors due to the presence of the fluid circulating across them. We, therefore, isolated the majority of the vIGT ion membrane from contact with this fluid (Fig. 1a). This design feature was possible because the de/doping of IGT channels occurs via internal ion reservoirs, and does not depend on ions from an external electrolyte^14,16,31^. However, water is still required for the effective conduction of ions within conducting polymers and the formation of the electric double layer^32^. As a result, the IGT channel must be hydrated for operation. We accomplished this channel hydration by introducing a micrometre-scale conduit–the H-via–that permits the transport of water from the external environment into the transistor channel.

To test these theoretical notions, we investigated the properties of the H-via and its effect on vIGT operation. We found that the H-via established a large electrochemical impedance between the channel and the external electrolyte compared with architectures that directly expose the full channel to the electrolyte (Fig. 2a and Supplementary Fig. 3). This impedance appeared to act as a large series electrochemical attenuator that minimized the spread of the gate potential into the electrolyte. Therefore, no signal (electrochemical or biological) was propagated across the H-via. Despite the presence of this large electrochemical impedance, the H-via did not alter the transfer curve of the transistor (Fig. 2b and Supplementary Fig. 4). Taken together, these findings confirm that the H-via does not serve as a substantive source for ions to operate the transistor; in this case, its high electrochemical impedance would deteriorate the transistor performance. Instead, the H-via permits the osmotic movement of water to hydrate the channel, but mobile ions internal to the channel material mediate the transistor operation, in line with previous mechanistic investigations of horizontal IGTs^14,16^.

We next aimed to determine whether the vIGT design was effective in eliminating crosstalk in an ion-rich environment. We fabricated vIGTs with different intertransistor spacing and monitored the crosstalk by observing the drain current of a biased transistor (T_2_; red) and operating its adjacent transistor (T_1_; black) using a squared gate voltage (Fig. 2c,d). There was no observable leakage at any pitch size achievable with contact lithography (Fig. 2e). Critically, the vIGT time constant was maintained across all the multitransistor configurations (Supplementary Fig. 5). Last, the vIGT equipped with the H-via exhibited consistent modulation for over one year, emphasizing the chemical stability of the channel material and the persistent functionality of the H-via (Fig. 2f and Supplementary Fig. 6). Therefore, the H-via enables stable, long-term and crosstalk-free operation of densely packed vIGTs.

By miniaturizing the channel through vertical stacking (Supplementary Fig. 7), the channel contact area becomes larger than the channel itself. This ratio of component sizes could potentially deteriorate the temporal response of the vIGT by forming parasitic capacitances^33,34^. Therefore, we investigated the temporal response of these devices as a function of their contact area. We first modelled the contacts as a series resistance (*R*_C_) and a parallel resistive-capacitive (*R*_P_, *C*_C_) circuit between the Au-based ohmic contacts of the drain and gate. A similar approach was taken for the gate interface (*R*_G_, *C*_G_). Fitting the electrochemical impedance spectroscopy measurements of these contacts revealed that the value of *R*_P_ was several orders of magnitude larger than the series resistances and could be neglected for the derivation of total resistance and capacitance (Fig. 3a)^35,36^. In this arrangement, the time constant also depends on the magnitude of the contact area relative to the gate area. If the gate area was larger than the contact area (*C*_G_ > *C*_C_), the total capacitance was controlled by the contact capacitance (Fig. 3b and Supplementary Fig. 8 (red)). Increasing the contact area in this regime gave rise to an overall increase in the time constant (↑*τ* = (*R*_G_ + *R*_C_)*C*_C_↑). If the contact area was larger than the gate area (*C*_G_ < *C*_C_), the overall capacitance was dictated by the gate capacitance (Fig. 3b and Supplementary Fig. 8 (blue)). In this case, increasing the contact area lowered the overall resistance of the circuit and enabled a faster time constant (↓*τ* = ↓(*R*_G_ + *R*_C_)*C*_G_). This set of relationships is only valid when the temporal response of the channel itself is substantially smaller than that of the contacts. Because IGTs rely on internal mobile ions for operation, the temporal response of the channel is dictated by hole mobility rather than ion mobility and the ensuing fast channel dynamics support the applicability of these parameters (Supplementary Fig. 9) ^37,38^. We then leveraged the vIGT design to increase the contact area and maintain a proportionally smaller gate. This modification led to a decrease in the overall response time with no substantial effect on transconductance (Fig. 3c and Supplementary Figs. 10 and 11). Additionally, there is a trade-off between the large contact area and the OFF current of the transistor, which could affect the overall ON/OFF ratio, a critical property for establishing digital electronics. This effect occurs because IGTs (similar to organic electrochemical transistors) are able to completely dedope the PEDOT:PSS channel of electronic carriers. As such, the OFF current is equal to the ionic drift current across the source and drain contacts, which is governed by *V*_D_ and the electrochemical impedance of the contacts. The smaller the contact area, the larger is the impedance and hence the smaller is the OFF current (Fig. 3d).

Given these properties of individual vIGTs, we hypothesized that it would be possible to generate large, conformable vIGT arrays with high transistor density. Using the scalable vIGT fabrication process with its intrinsic multilayer metallization, we created a 3-μm-thick conformable integrated circuit with ~155,000 transistors cm^−2^ density in a common-source matrix structure comprising a total of half a million transistors (Fig. 4a,b). This transistor density surpasses other flexible transistors, including those with high-throughput production capacity and photopatternable semiconducting channels (Fig. 4c). We next demonstrated the functionality of vIGTs by developing several circuit components. Because physiologic signals are often low amplitude, we created a multistage, high-speed amplifier with a gain exceeding 650 at an operating voltage of 600 mV to facilitate safe, effective amplification even in direct contact with biologic tissue (Fig. 4d). To highlight the consistency and precision of vIGT fabrication, we also generated a ring oscillator at 2.5 kHz (Fig. 4e). Multiplexers are key components of multichannel acquisition devices because they increase the number of signals that can be sampled relative to the number of interconnects. The vIGTs formed high-performance multiplexing switches with low crosstalk (less than −60 dB; Fig. 4f). Another advantage of vIGTs is the ability to control the reduction state of the channel, permitting the creation of diodes and rectifying circuitry using a single channel material (Fig. 4g, top). The threshold voltage (*V*_t_) of these organic diodes could be tuned from −0.2 to 0.2 V depending on the concentration of PEI in the PEDOT:PSS channel, which is substantially lower than the hydrolysis potential and *V*_t_ of Si-based diodes (Fig. 4g; bottom).

The combination of high-speed, efficient ion-to-electron conversion and low operating voltage characteristics of vIGTs opened unique possibilities for device integration. We hypothesized that we could create a fully conformable, stand-alone vIGT-based bioelectronic implant that acquires and amplifies physiologic data, wirelessly transmits these data to the external environment and operates via wireless power^22^. To accomplish this, we needed to power a vIGT using fast alternating current (a.c.) rather than direct current (d.c.). It has been previously shown that high-frequency electrical signals applied between a pair of contacts efficiently propagate through ion-rich media, including intact biological tissue^20,39^. Based on this concept, it would be theoretically possible to bias an implanted transistor by electric potentials applied non-invasively at a distance. Because vIGTs can operate at high frequencies, we could bias the transistor at a desired set point once per cycle of the applied a.c. waveform. For example, a 500-mV-amplitude, 1-MHz-frequency sine wave would set the channel potential to −500 mV at the trough of each cycle (which occurs every microsecond). In turn, the vIGT amplifies the physiologic activity at its gate (input) and modulates the amplitude of the received sine wave to encode this signal. The amplified and modulated sine wave can then be transmitted back across the ion-rich medium using a third contact. This signal is received and differentially amplified with respect to the initially transmitted sine wave to permit the extraction of the physiologic signal (Fig. 5a).

Because the power was transmitted to the vIGT via alternating potential applied across two distant power contacts, the geometry of these contacts was critical. With the application of 500 mV across the contacts, we found that approximately 500 μA mm^−2^ normalized current per power contact area was delivered to the vIGT (Fig. 5b,c). This current capacity directly translates into the drain current of the vIGT and is on par with typical *I*_D_ values in the d.c. operation mode. Thus, ionic communication (IC) can supply sufficient power to operate vIGTs. Next, we investigated the range of carrier frequencies over which the vIGTs could maintain amplification. We previously established that the effective carrier frequency range for IC is between 50 kHz and 5 MHz in biological tissue. The vIGT demonstrated consistent current modulation and voltage gain (ratio of input/output voltage) across this frequency range, and values were similar to those obtained via the application of d.c. potentials (Fig. 5d,e).

To test these concepts in a realistic setting, we developed a stand-alone device that utilized a vIGT to acquire and amplify neurophysiologic signals, and IC to power the vIGT and transmit data to the external environment. (Fig. 6a). The transistor was configured as a common-source amplifier. The two conducting polymer-based power contacts were placed on the surface of the rat’s skull and the output potential was extracted from the node between the drain and power contact by a third conducting polymer-based electrode (the data contact). Interestingly, we found that the power contact effectively served as the load for the vIGT-based amplifier in this circuit design. A matching three-contact array was fabricated and aligned over the intact tissue interface to transmit power and data (Supplementary Fig. 12). We generated somatosensory evoked potentials (SSEPs) by electrical stimulation of the hindlimb and compared the responses obtained by a vIGT shank coupled to the IC arrays and a conventional neural probe attached to Si-based amplifiers with cables for data transmission. The vIGT-based device was able to acquire the SSEPs with similar signal-to-noise ratios to the conventional device, and accurately conveyed the known relationship between the applied current and SSEP amplitude (Fig. 6b,c). We then monitored the performance of these devices in a chronic setup. In freely moving rats, we recorded spontaneous neural activity across behavioural-state transitions (Supplementary Fig. 13). Furthermore, the power spectra from the vIGT-based device and the conventional device were not significantly different, highlighting the ability of the vIGT-based device to accurately sample and transmit neurophysiologic data (Fig. 6d,e). Because the stand-alone device relies on megahertz-range frequencies for powering and data transmission, we quantified the maximum voltages applied to the tissue as a function of transmitter electrode geometry and device operating voltage (Supplementary Fig. 14). These values were well within the safety boundaries for induced electric fields in tissue as delineated by the International Commission on Non-Ionizing Radiation Protection.

Bioelectronics are increasingly required to perform complex signal processing. The current approach involves the encapsulation of active electronics in rigid enclosures, increasing the physical footprint of the device and the complexity of the implantation procedure. Here vIGTs offer safe, effective signal processing functions in the presence of physiologic media, removing the need for a barrier between the active bioelectronic device and tissue. These benefits are of particular relevance for devices targeting sensitive, electrically active tissue, such as the brain. In addition, applications that interface with geometrically complex or dynamic structures (spinal cord^40,41^, subdermal tissue^42,43^ and peripheral nerve^44,45^) can benefit from the conformable vIGT-based circuitry. Because the stand-alone vIGT components are miniaturized and show high performance, they preserve the ability to sample and process signals at high spatiotemporal resolution. Conventional neural interface devices encounter substantial limitations of channel count, sampling rate and amount of data that can be stored and/or transmitted (such as NeuroPace^46^). The limitations arise mostly due to the physical footprint and properties of the components used. Furthermore, vIGTs offer the potential to increase the amount of data that can be safely acquired and processed using implantable devices, in turn enhancing the precision of diagnostics and therapeutics enabled by such devices^5,21^.

We have shown that vIGTs broaden the domain of organic electronics from acquisition to advanced processing and signal/power transmission. As a result, vIGTs enabled the creation of the first fully organic, conformable, stand-alone neural interface device. Such advances could decrease the risks of bioelectronic devices without compromising performance, leading to novel, more accessible applications that benefit human health.

## Methods

### Material preparation

PEDOT:PSS (Clevios PH1000) was purchased from Heraeus. Chitosan (low molecular weight), d-sorbitol (≥99.5%; BioUltra), (3-glycidyloxypropyl)trimethoxysilane, 4-dodecyl benzene sulfonic acid, 3-(trimethoxysilyl)propyl methacrylate (A-174 silane), branched PEI and acetic acid were purchased from Sigma-Aldrich. Micro-90 concentrated cleaning solution was purchased from Special Coating Services. AZ nLOF 2020 (negative photoresist), AZ 10XT (positive photoresist), AZ 400K and AZ 300 MIF (metal ion free) developers were acquired from MicroChemicals, Merck. To create the transistor channel, a mixture of PEDOT:PSS aqueous dispersion and d-sorbitol (40 wt%) was prepared and mixed with (3-glycidyloxypropyl)trimethoxysilane (1.0 wt%) and 4-dodecyl benzene sulfonic acid (0.1 wt%). PEI was diluted in deionized water (1:10). Chitosan (0.5 wt%) was diluted in deionized water and mixed with acetic acid (6.0 wt%).

### Device fabrication

Silicon wafers (outer diameter, 100 mm; thickness, 500 μm; SSP) were coated with 1.8 μm of parylene C through chemical vapour deposition using SCS Labcoter 2. Metal contacts and interconnects were patterned through three separate metal lift-off processes. The first metal lift-off was performed to pattern the layout for the drain contacts and interconnects. AZ nLOF 2020 photoresist was spin coated at 3,000 r.p.m. on the substrate, baked at 110 °C for 90 s for both soft and post-exposure bake, exposed to ultraviolet light using a Suss MA6 mask aligner and developed with AZ 300 MIF for 2 min. A 10-nm-thick Ti adhesion layer followed by a 150-nm-thick Au layer were deposited using an electron-beam evaporator (Angstrom EvoVac multiprocess evaporator) and patterned by soaking the substrate in a bath of resist remover. The resulting patterned metal layer was insulated with 400 nm of parylene C. The adhesion between the first and second parylene C layer was enhanced by the addition of A-174 silane during the chemical vapour deposition. A second metal lift-off was performed to create gate contacts and H-via etch stop contacts, followed by PEDOT:PSS and ion membrane patterning. To pattern the PEDOT:PSS, a peel-off technique was used. To facilitate the peel off, an anti-adhesion agent (5 wt% Micro-90 diluted in deionized water) was spin coated at 1,500 r.p.m before the deposition of a 1.8 μm parylene C layer. Parylene C was patterned with a 4.6-μm-thick AZ9260 photoresist and dry etched with a plasma reactive ion etching process (Oxford Plasmalab 80; 180 W, 50 s.c.c.m. O_2_ and 2 s.c.c.m. SF_6_). Also, AZ9260 was spin coated at 5,000 r.p.m., baked at 110 °C for 90 s, exposed using a Suss MA6 mask aligner and developed with AZ 400K developer (1:4 with deionized water). PEDOT:PSS was spin coated at 1,000 r.p.m. and peeling off was performed. Subsequently, chitosan was spin coated at 1,500 r.p.m. and baked at 120 °C for 5 min to create a homogeneous ion membrane film. A thin parylene C layer (<150 nm) was deposited and patterned through photolithography using 4.6-μm-thick AZ9260 photoresist and dry etched with a plasma reactive ion etching process in a similar manner as the one described above to shape the ion membrane on the gate contact (gold/PEDOT:PSS). An additional 400 nm of parylene C was deposited, adding A-174 silane in the chamber where the chemical vapour deposition process takes place to enhance adhesion. A third metal lift-off process was performed for the creation of the source electrodes and interconnects followed by two parylene C depositions of 1.8 μm each. The first 1.8-μm-thick parylene C layer was used as an insulation layer for the transistor structure and A-174 silane was employed during the deposition to enhance adhesion with the underlying layer. The first 1.8-μm-thick parylene C layer served as a sacrificial layer for the subsequent peel-off process. To facilitate the peel off, an anti-adhesion agent (5 wt% Micro-90 diluted in deionized water) was spin coated at 1,500 r.p.m between the two parylene C layers. The stacked layers were patterned using a Ti-based hard-etch mask and dry etched with a plasma reactive ion etching for defining the transistor channel area and electrical contact pads on top of the peelable sacrificial parylene C layer. Note that the source and drain contacts serve as etch stops. The transistor channels were created by spin coating PEDOT:PSS (5,000 r.p.m.) for depletion-mode IGTs with the addition of a PEI layer (1,000 r.p.m.) for enhancement-mode IGTs and patterned by peeling off the sacrificial parylene C layer. The transistors were insulated by an additional 2.5-μm-thick parylene C layer and their channel hydration was created through a vertical conduit (H-via) connecting the ion membrane with the external environment. H-vias and electrical contact pads were opened using dry etching and patterned via photolithography using the AZ 9260 photoresist.

### Electrical characterization

The *I*–*V* characteristics were measured with a Keysight B2902A precision source measurement unit (SMU) using two channels and its associated measurement software (Keysight BenchVue 2019 and B2900 Quick IV 4.2.2045.2760). The first channel supplied the drain voltage (*V*_D_) and measured the drain current (*I*_D_), whereas the second channel provided the gate voltage (*V*_G_). Temporal responses were recorded with an oscilloscope (Keysight InfiniiVision EDUX1002A). The gate voltage pulses were supplied by a function generator (Keysight 33500B series), whereas *V*_D_ was provided by an SMU (Keysight B2902A). The *I*_D_ value was derived by measuring the voltage fluctuations across a 100 Ω resistor in series with the transistor channel (common-source configuration). The resulting drain current curve was fitted with a single exponential decay equation to extract the time constant of the transistors. For multiplexer measurements, the input sine wave and gate pulses were supplied by two separate function generators (Keysight 33500B series), whereas the output was recorded with an oscilloscope (Keysight InfiniiVision EDUX1002A). The channel mobility was measured using previously established time-of-flight measurements by deriving the transients of drain current under the application of various constant gate currents^47^.

#### Diode-connected vIGT characterization.

The *I*–*V* curves of diode-connected vIGTs were measured with a Keysight B2902A precision SMU. Here *I*_D_ was measured with *V*_D_ swept from −0.6 to 0.6 V. For the temporal response measurement, *V*_D_ was supplied with a series of sine waves across the frequency bandwidth using a function generator, with a current amplifier (TSC213, STMicroelectronics) used to measure *I*_D_.

#### Gain characterization of vIGT.

An SMU was used to measure the vIGT voltage gain. Here *V*_G_ and *I*_D_ were supplied by two SMU channels. Also, *V*_D_ was measured with *V*_G_ swept from −0.6 to 0.6 V with a logarithmic sweep of *I*_D_. The sweep rate was set to 300 mV s^−1^ for the output characteristics measurements. For the characterization of cascaded vIGTs, *V*_D_ of the first vIGT was connected to the gate of the second vIGT. Also, *I*_D_ of the second IGT was supplied by a third SMU channel. To test the gain of vIGTs with an a.c. supply, *V*_OUT_ of a vIGT common-source amplifier was measured with *V*_D_ supplied with a sine wave and *V*_G_ supplied with a waveform generator (Keysight 33500B series).

#### IC power delivery characterization.

IC contacts (2.5 × 2.5 mm^2^) were fabricated out of gold. The transmitter contacts were connected to a signal generator (Keysight 33500B Series). The amplitude of the received signal was measured with a battery-powered oscilloscope (Micsig TO1104) with/without a load resistor. The impedance of the IC contacts was derived using the following equation.


$$R_{\text{IC}}=\left(\frac{V_{\text{Open}}}{V_{\text{Closed}}}-1\right)\times R_{\text{Load}}$$

#### A vIGT multiplexer.

The vIGT multiplexer performance was characterized with a dual-channel waveform generator (Keysight 33500B series). A 100 mV, 100 Hz sine wave with −0.6 V bias was applied as *V*_D_. A 10 kHz square wave alternating from −0.6 to 0 V with 50% duty cycle was sourced to *V*_G_. The output was recorded with an oscilloscope (Keysight InfiniiVision EDUX1002A).

### Electrochemical impedance

Electrochemical impedance spectra, modelling and equivalent circuit extraction were performed with a Gamry Reference 600+ instrument using Ag/AgCl and Pt electrodes as the reference and counter electrodes, respectively. The native Gamry firmware was used to conduct the experiments. Electrochemical impedance was measured in the potentiostatic mode with 100 mV_r.m.s._ and frequency range from 1 Hz to 5 MHz (10 points per decade). For H-via impedance spectroscopy, a four-electrode setup was implemented to isolate the measurement of what is in between the pairs of electrodes (in this case, the H-via), independent of the electrochemical impedance of the contacts. Calibration of four-electrode potentiostatic-mode electrochemical impedance spectra was done using a ceramic-based 50 MΩ resistor. Phosphate-buffered saline was used as the electrolyte in all the impedance measurements.

### IGT-based wireless neural implant

#### External module.

A battery-powered dual-channel signal generator (Koolertron, DDS Signal Generator) was used to provide power to the vIGT stand-alone device (2 V, 1 MHz) and provide the common-mode rejection signal to suppress the carrier wave before the amplification stage. The external receiver was designed to extract the envelope of the amplitude-modulated signal. This system consisted of an active bandpass filter (50–150 kHz; Texas Instruments, OPA2320), a high-bandwidth variable-gain amplifier (Analog Devices, AD8338), an envelope detector (Analog Devices, ADL5511) and a custom voltage amplifier with an active bandpass filter (Texas Instruments, OPA2320). For each recording, the phase-locked common-mode rejection signal was fine-tuned to cancel the carrier wave from the amplified signal. The recovered electrophysiologic signals were recorded with an analogue-to-digital converter (AD7680). The operation of the external module was validated using a lock-in amplifier (Stanford Research Instruments, SR830).

### Animal surgical procedure

All the animal experiments were approved by the Institutional Animal Care and Use Committee at Columbia University. Two male Long–Evans rats (200–350 g, 8–15 weeks of age) were used for intracranial implantation. The rats were kept on a regular 12 h–12 h light–dark cycle and housed in pairs before implantation, but separated afterwards. No prior experimentation had been performed on the rats.

### SSEP recording

The rats were initially anaesthetized with 2.00% isoflurane and maintained under anaesthesia with 0.75–1.00% isoflurane during the surgery. To minimize brain swelling and inflammation, methylprednisolone (30 mg kg^−1^) was administered during surgery. A 3 × 3 mm^2^ cranial window over the somatosensory cortex (anterior-posterior (AP), 2 mm; medial-lateral (ML), 3 mm) was opened and the dura mater was removed. Neural probes were placed on the cortical surface to record SSEPs. The wireless power/communication pad was placed on top of the skull. The surgery site was sutured after the surgery. A pair of stainless steel needle electrodes was inserted 5 mm apart into the muscle under the skin of the hindpaw of the rat to provide peripheral electrical stimulation. Electrical stimulation was applied with a constant-current stimulus isolator (World Precision Instruments, A365RC). Stimulation was controlled by a battery-powered microcontroller (STMicroelectronics, STML432KC), with 100 μs pulse width and 5 s interval between individual pulses. The stimulation intensity was incremented from 1 to 6 mA. The SSEPs were recorded first with a 128 channel NeuroGrid^48^ and subsequently with the vIGT stand-alone device.

### Chronic implantation

The rats were initially anaesthetized with 2.00% isoflurane and maintained under anaesthesia with 0.75–1.00% isoflurane during the surgery. To minimize brain swelling and inflammation, methylprednisolone (30 mg kg^−1^) was administered during surgery. A 3 × 3 mm^2^ cranial window over the hippocampus (AP, 3.5 mm; ML, 3.5 mm) was opened and the dura mater was removed. The vIGT shank was inserted to a depth of −2.5 mm dorsal-ventral (DV) using a chemically polished 50 μm tungsten wire as a guide. The wireless power/communication pad was placed on top of the skull. After implantation, the craniotomy was covered with gel foam and sealed using a silicone elastomer. All the surgical incisions were sutured closed.

### In vivo electrophysiology recording

In vivo recording was performed both during anaesthesia and with the rat freely moving. The receiver array was aligned with the implanted contacts and placed on the surface of the skin of the scalp. Electrophysiologic signals were whitened and the spectra were calculated in MATLAB 2021b (MathWorks). We used custom MATLAB code to score the behaviour into rapid-eye-movement (REM) sleep, non-REM sleep and waking epochs based on the spectral features and an onboard accelerometer. The spectral analyses were generated using a Gabor-based analytical wavelet.

### Reporting summary

Further information on research design is available in the Nature Portfolio Reporting Summary linked to this article.

## Supplementary Material

SI

2

## Figures and Tables

**Fig. 1 |:**
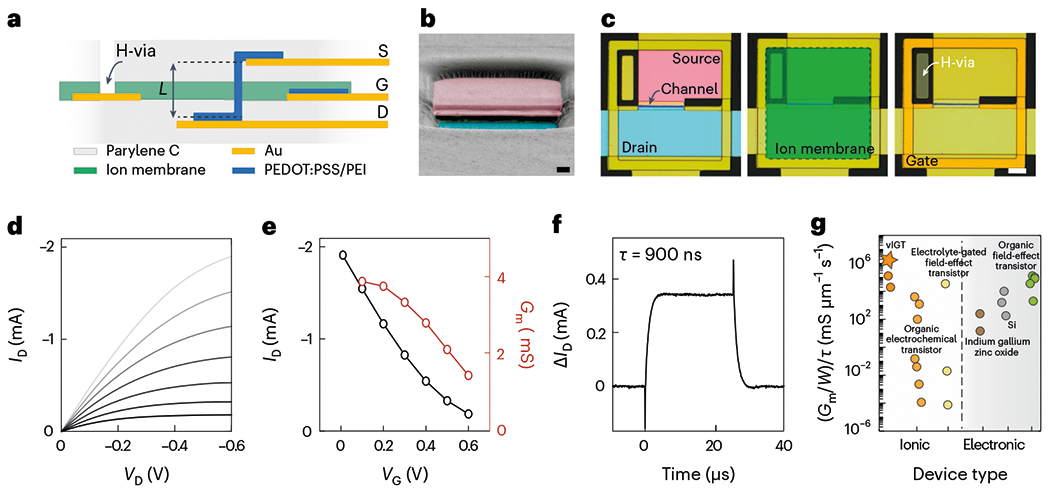
Physical structure and electrical characteristics of vIGTs. **a**, Schematic of a vIGT cross section consisting of a vertical channel length (*L*) defined by the thickness of the interlayers between the source (S) and drain (D) contacts. The H-via was a micro-conduit from the surface of the device through the ion membrane layer to permit channel hydration. G, gate. **b**, Colourized cross-section scanning electron microscopy image of a vIGT after defining the channel area. The pink and blue regions are the source and drain contacts, respectively. Scale bar, 800 nm. **c**, Optical micrograph displaying the top view of an individual vIGT. Each part of the transistor is highlighted with a different colour (blue, drain contact; pink, source contact; ion membrane, green; H-via, dark green). Scale bar, 5 μm. Note that the PEDOT:PSS that is interposed between the Au of the gate and the ion membrane is not visible in this view. **d**, Output characteristics of a depletion-mode vIGT device (*W/L* = 5.0/0.8 μm; thickness, *d* = 100 nm) for *V*_G_ varying from 0 V (top curve) to 0.6 V (bottom curve) with a step of 0.1 V. **e**, Transfer and transconductance curves extracted from the output characteristics for *V*_D_ = −0.6 V. **f**, Corresponding temporal response of the drain current (*I*_D_) for *V*_D_ = −0.6 V and *V*_G_ pulse amplitude of 0.1 V; an exponential fit of the vIGT drain current resulted in a time constant of 0.9 μs (*n* = 128 pulses per current measurement and *n* = 5 devices; Supplementary Figs. 2c and 11e). **g**, Performance of flexible transistors as characterized by the normalized ratio of transconductance and rise time versus channel width. Devices are categorized based on their architecture as well as ionic/electronic interaction. Supplementary Table 1 and Supplementary Fig. 2d–f provide details of the materials and devices. The star symbol notates the vIGT.

**Fig. 2 |:**
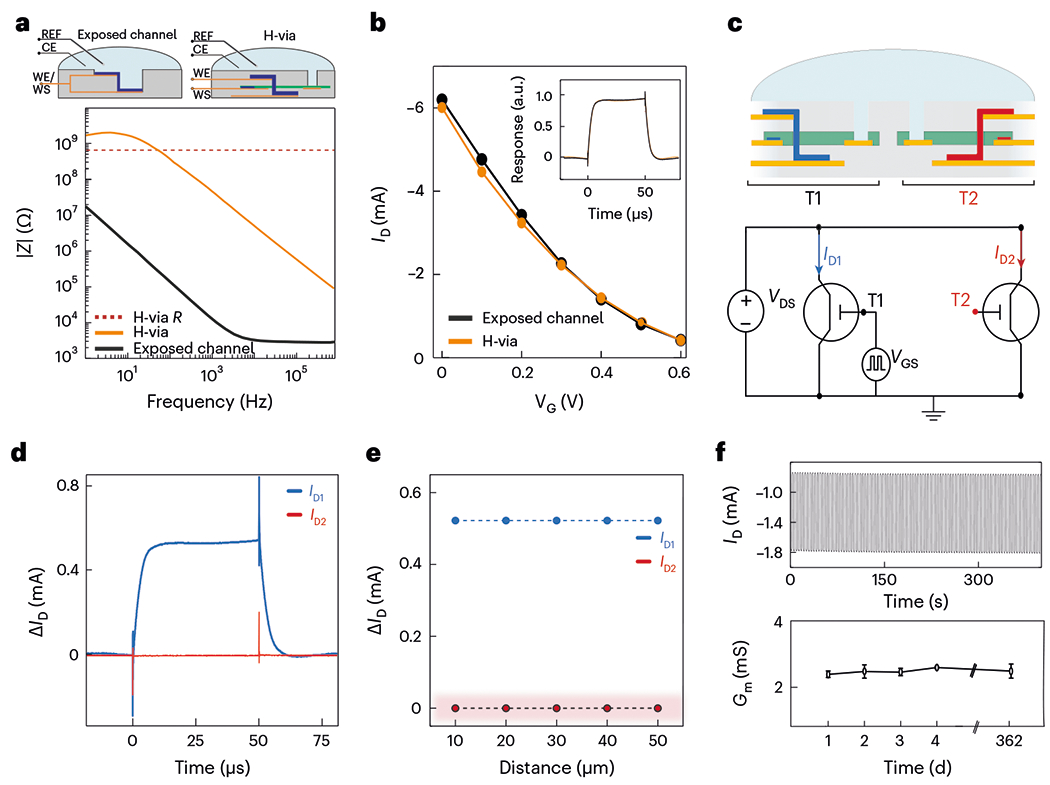
Crosstalk-free and stable operation of densely packed vIGTs in physiologic media. **a**, Electrochemical impedance spectra of vIGT (*W/L* = 5.0/0.8 μm, *d* = 200 nm)with the channel area exposed to the electrolyte and H-via (*R* = 752.50 ± 43.25 MΩ). The insets show the wiring diagrams of a vIGT with channel interfacing exposed to the electrolyte (left) and the H-via interfacing with the electrolyte (right). Methods provides the experimental details (H-via size = 5 × 5 μm^2^, *n* = 5 repetitions per data point per device; *n* = 4 devices). REF, reference electrode; CE, counter electrode;WE, working electrode;WS, working sense electrode. **b**, Transfer curves of vIGT with the full channel exposed to the electrolyte and vIGT with H-via exposure only demonstrating that the H-via does not impede vIGT performance (*W/L* = 5.0/0.8 μm, *d* = 100 nm, *V*_D_ = −0.6 V; *n* = 6 devices). **c**, Schematic of two adjacent vIGTs immersed in physiologic media. The H-via eliminates crosstalk between closely adjacent transistors (top). The wiring diagram of two adjacent vIGTs used for crosstalk characterization is shown; both transistors were powered and T_1_ received a pulsed voltage to *V*_G_, whereas T_2_ was monitored in the absence of an input to its own gate (bottom). **d**, Transient responses of drain currents *I*_D1_ and *I*_D2_ operating at *V*_D_ = −0.4 V, with pulsed *V*_GS_ between 0 and 0.6 V (*n* = 128 pulses per current measurement, *n* = 5 devices, *W/L* = 5.0/0.8 μm, *d* = 100 nm). **e**, Drain current modulation of an individual vIGT without inducing the crosstalk or modulation of neighbouring transistors at varying distances. Measurements were simultaneously obtained using operating parameters and device geometry as in **d**. The highlighted area indicates the limit of instrument sensitivity. Supplementary Fig. 5 shows the individual pulse waveforms. **f**, Sample temporal response of the drain current (*I*_D_) of the vIGT under continuous operation for 400 s (*n* = 400 pulses; top). The maximum transconductance (*G*_m_^max^) of a vIGT over a period of 362 days under continuous pulsed gate voltages (*V*_D_ = −0.4 V and *V*_G_ pulses from 0 to 0.4 V; same geometry as in **d**; bottom). Each point represents the average transconductance under continuous operation for 50 min (3,000 pulses). The error bars represent the standard deviation value (bottom). Day 1, 2.3925 ± 0.0419 mA; day 2, 2.4775 ± 0.1345 mA; day 3, 2.4450 ± 0.0443 mA; day 4, 2.5967 ± 0.0231 mA; day 362, 2.4500 mA ± 0.2970; mean ± standard deviation.

**Fig. 3 |:**
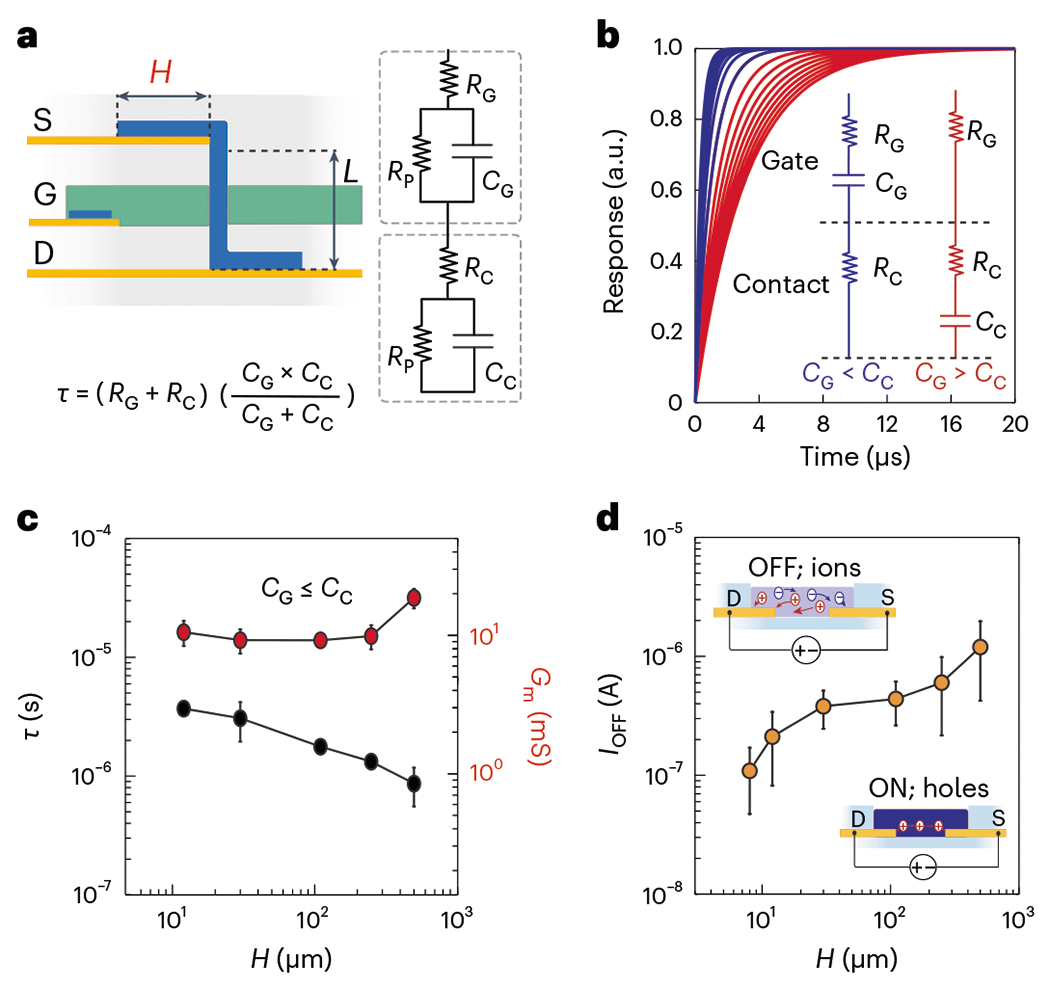
Contact length has nonlinear effects on the temporal response. **a**, Schematic of a vIGT displaying channel length (*L*) and contact length (*H*; left). A circuit diagram of the model showing the electrical characteristics of the gate (*R*_G_, *C*_G_) and channel contacts (*R*_C_, *C*_C_ and *R*_P_; right). Time constant (*τ*) equation of the series gate and contact circuits (*R*_P_ » *R*_G_, R_c_; bottom). **b**, Simulated transient behaviour of a vIGT in response to a square step of the gate voltage. If *C*_G_ > *C*_C_, the transient response depends only on *C*_C_, *R*_C_ and *R*_G_; hence, larger contact lengths result in a slower transient response. If *C*_G_ ≤ *C*_C_, the transient response depends on *C*_G_, *R*_G_ and *R*_C_; hence, larger contact lengths result in a faster transient response. **c**, Transient response of vIGTs with a range of contact lengths (*H*; spanning from smaller to larger than the gate area). *H* = 12 μm, *τ* = 3.70 ± 0.28 μs. *H* = 30 μm, *τ* = 3.00 ± 0.74 μs. *H* = 110 μm, *τ* = 1.70 ± 0.14 μs. *H* = 250 μm, *τ* = 1.30 ± 0.06 μs. *H* = 500 μm, *τ* = 0.80 ± 0.20 μs. The vIGT transconductance showed a small increase with the largest contact lengths. *H* = 12 μm, *G*_m_ = 10.54 ± 1.45 mS. *H* = 30 μm, *G*_m_ = 9.23 ± 1.22 mS. *H* = 110 μm, *G*_m_ = 9.22 ± 0.48 mS. *H* = 250 μm, *G*_m_ = 9.88 ± 1.30 mS. *H* = 500 μm, *G*_m_ = 18.67 ± 1.90 mS. Statistics are reported as mean ± standard deviation (*n* = 3 devices per contact length). **d**, OFF current of vIGT was defined by the impedance and geometry of the contact lengths (*H*; schematic in the inset). An increasing contact area results in a larger OFF current. *H* = 8 μm, *I*_OFF_ = 1.09 × 10^−9^ ± 6.16 × 10^−8^ A. *H* = 12 μm, *I*_OFF_ = 2.11 × 10^−7^ ± 1.29 × 10^−7^ A. *H* = 30 μm, *I*_OFF_ = 3.80 × 10^−7^ ± 1.34 × 10^−7^ A. *H* = 110 μm, *I*_OFF_ = 4.38 × 10^−7^ ± 1.75 × 10^−7^ A. *H* = 250 μm, *I*_OFF_ = 6.01 × 10^−7^ ± 3.84 × 10^−7^ A. *H* = 500 μm, *I*_OFF_ = 1.60 × 10^−6^ ± 4.45 × 10^−7^ A. Statistics are reported as mean ± standard deviation (*n* = 3 devices per contact length).

**Fig. 4 |:**
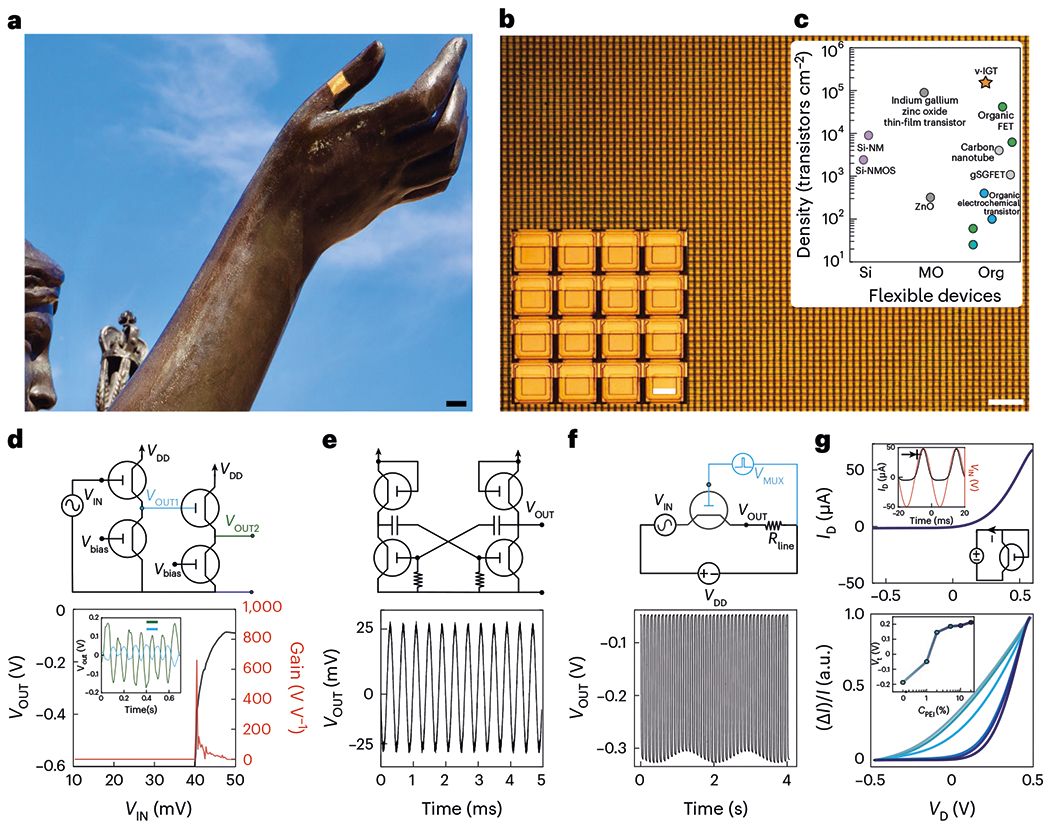
Conformable, high-density vIGT-based integrated circuits. **a**, A 3-μm-thick vIGT array conforming to a complex curvilinear surface. Scale bar, 10 mm. **b**, Optical micrograph of a conformable vIGT array with a density of 155,586 transistors cm^−2^. Scale bar, 30 μm. The inset shows a magnified image of the vIGT array. Scale bar, 4 μm. **c**, Fabrication density of flexible transistors based on channel material (Si, silicon; MO, metal oxide; Si-NMOS, silicon n-type MOS; Org, organic; Supplementary Table 2 provides the device details). **d**, Circuit diagram of an active-load, multistage, cascaded vIGT-based inverter (top) with the corresponding input/output and gain (*G* = 650; bottom). The inset shows the application of the circuit as a voltage amplifier for 25 μV_PP_ sine-wave input signals. **e**, Astable multivibrator oscillator constructed by two active-load vIGT inverters (top). The output voltage of the vIGT-based oscillator operating at 2.5 kHz (bottom). **f**, Circuit diagram of a vIGT-based multiplexer switch (top) and the corresponding output signal of the switch performing time-division multiplexing of a sine-wave input signal (bottom; *V*_D_ = −0.6 V and *V*_G_ = 100 mV_PP_). **g**, Current–voltage (*I*–*V*) characteristics of the vIGT operating as a diode. The insets demonstrate the vIGT as a diode-connected transistor rectifying a sine wave (top). Superimposed *I*–*V* characteristics of a vIGT configured as a diode-connected transistor with different threshold voltages. The darker colours represent a high concentration of PEI; the inset shows the modulation of the threshold voltage of a diode-connected transistor as a function of PEI concentration (bottom).

**Fig. 5 |:**
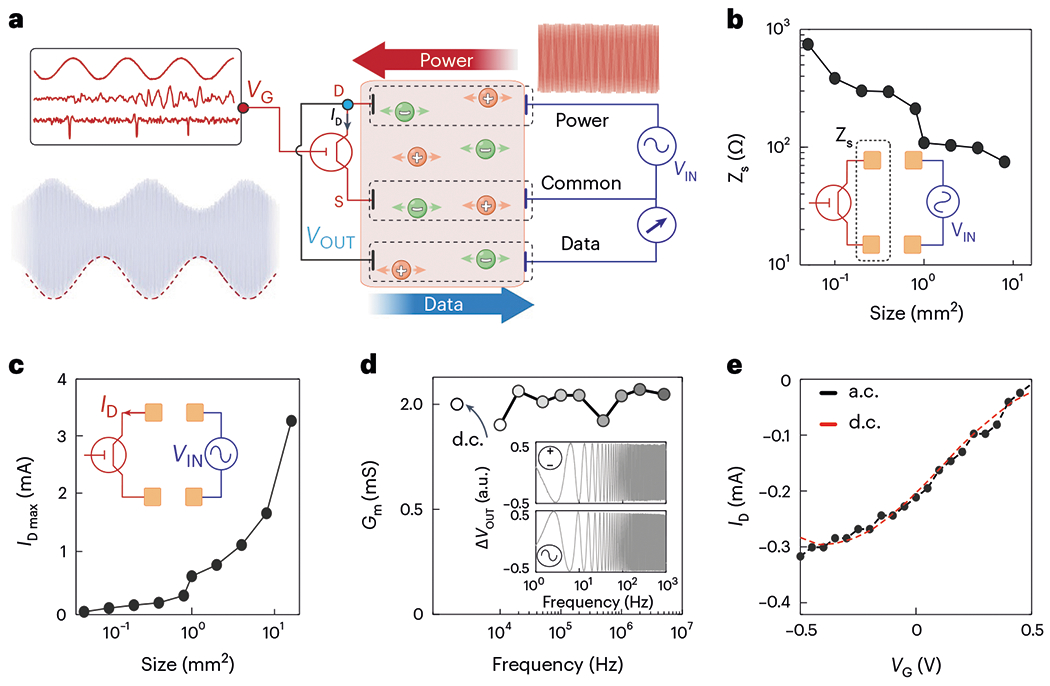
Integration of vIGT with IC establishes a fully conformable, stand-alone device with wireless power and data transmission. **a**, Schematic of working principles permitting vIGT powering via an alternating sine wave transmitted across a physiologic medium using two sets of aligned power contacts (power and common). The vIGT modulates this sine wave according to the electrophysiologic signal at its gate. This encoded signal is then routed to the data contact for transmission back across the medium. At the receiver, the data are acquired and demodulated with respect to the common electrode potential to decode the electrophysiologic signal. **b**, Two-terminal electrochemical impedance (at 100 kHz) varies relative to the geometry of the power contacts as a function of data and common electrodes. These impedance values correspond to the overall power source impedance (*Z*_s_) at each geometry. The inset illustrates the arrangement of IC power electrode pairs. **c**, Maximum current delivery capacity varies relative to the power contact size (*V*_DS_ = 500 mV_PP_). This current represents the maximum possible *I*_D_ that can be delivered to the vIGT via IC at each geometry. **d**, Transconductance values of vIGTs are stable across a wide range of carrier frequencies up to 5 MHz and are consistent with d.c.-powered values. The inset demonstrates the consistent sweep response of a vIGT when sourced with d.c. (*V*_DS_ = −500 mV) and a.c. (*V*_DS_ = −500 mV_pp_; 1 MHz) power. **e**, Transfer curves of the vIGT are similar when sourced with d.c. (*V*_DS_ = −500 mV) and a.c. (*V*_DS_ = −500 mV_pp_; 1 MHz) power.

**Fig. 6 |:**
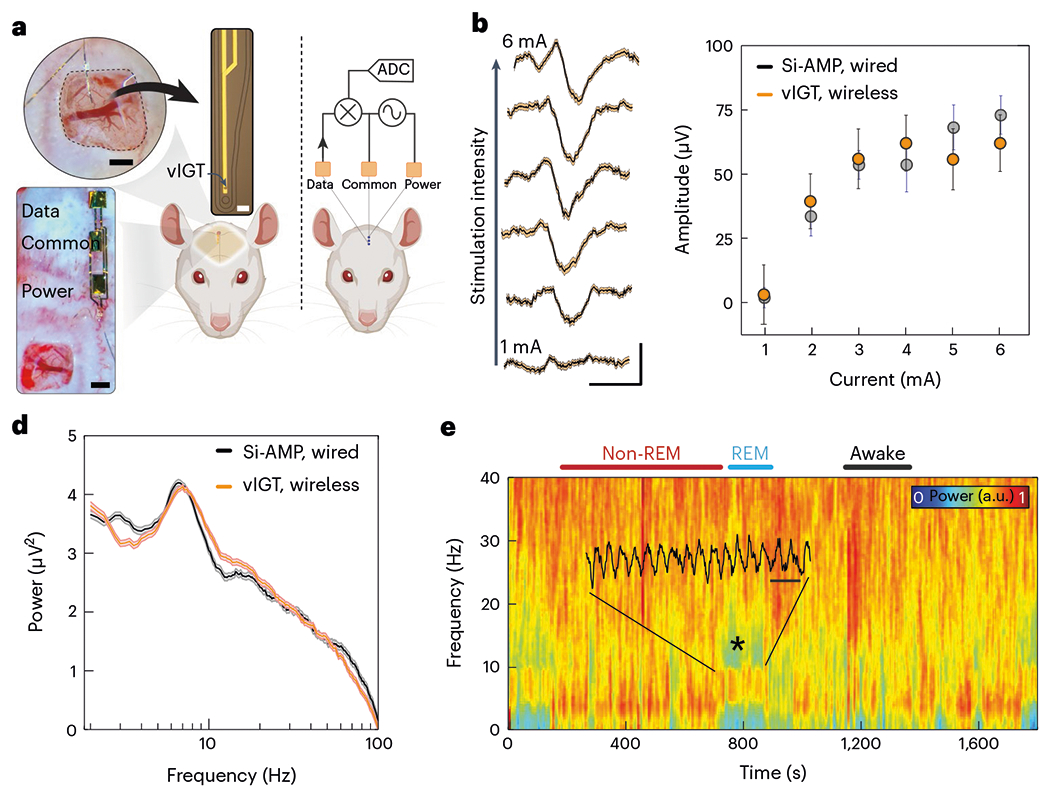
Fully conformable, implanted, vIGT-based stand-alone device performs in vivo acquisition and wireless signal transmission of neurophysiologic activity. **a**, Micrograph of a conformable vIGT-based neural shank being placed on the somatosensory cortex (top left). Scale bar, 500 μm. A micrograph of a vIGT-based shank and its interconnects (top right). Scale bar, 20 μm. A micrograph of the power, data and common contacts laminated on the surface of the skull (bottom left). Scale bar, 500 μm. Schematic of the power generator and data receiver electrodes placed on top of the scalp (right). ADC, analogue-to-digital converter. **b**, SSEPs recorded and wirelessly transmitted using a vIGT-based stand-alone device as the peripheral stimulation intensity is increased from 1 to 6 mA (bottom to top). Scale bar, 20 ms, 50 μV; traces and shaded error bars show the mean ± standard deviation; *n* = 120 trials. Si-AMP, silicon-based amplifier. **c**, Relationship between peripherally applied stimulation current and SSEP amplitude for a vIGT-based stand-alone device (1 mA, 5.850 ± 1.780 μV (*n* = 95); 2 mA, 38.160 ± 6.150 μV (*n* = 73); 3 mA, 54.730 ± 7.780 μV (*n* = 54); 4 mA, 60.850 ± 6.670 μV (*n* = 64); 5 mA, 54.510 ± 7.590 μV (*n* = 59); *I* = 6 mA, 60.810 ± 6.600 μV (*n* = 67 trials)) and conventional neural interface device (1 mA, 0.608 ± 3.920 μV (*n* = 25); 2 mA, 32.310 ± 6.180 μV (*n* = 37); 3 mA, 52.310 ± 5.870 μV (*n* = 22); 4 mA, 52.420 ± 9.890 μV (*n* = 28); 5 mA, 66.950 ± 6.360 μV (*n* = 46); 6 mA, 71.770 ± 7.390 μV (*n* = 37)). Statistics are reported as mean ± standard deviation and *n* represents the trial count. **d**, Power spectra extracted from an epoch of REM sleep by vIGT-based standalone device and conventional neural interface device. Statistics in the form of shaded error bars are reported as mean ± standard deviation (*n* = 200 trials). **e**, Time–frequency spectrogram of the neural data acquired and wirelessly transmitted using a vIGT-based stand-alone device demonstrates the characteristic local field potential (LFP) patterns corresponding to wakefulness, REM sleep and non-REM sleep. The superimposed raw time trace highlights the theta oscillations during REM sleep. Scale bar, 250 ms.

## Data Availability

All data needed to evaluate the conclusions in this study are provided in the Article and/or its Supplementary Information. All source files and experimental data are freely and publicly available at https://www.dion.ee.columbia.edu/ and via the public repository at https://doi.org/10.17632/5yjgb8pt4r.1.
