# Supplementary material for: Integrated internal ion-gated organic electrochemical transistors for stand-alone conformable bioelectronics
Source: Nat Mater. Author manuscript; Available in PMC 2023 Oct 2. (PMC10533388; doi:10.1038/s41563-023-01599-w)
Supplement: SI [file NIHMS1922635-supplement-SI.pdf]

nature materials

Article https://doi.org/10.1038/s41563-023-01599-w

# Integrated internal ion-gated organic electrochemical transistors for stand-alone conformable bioelectronics

In the format provided by the authors and unedited

# Integrated internal ion-gated organic electrochemical transistors for stand-alone conformable bioelectronics

Claudia Cea[†1], Zifang Zhao[†1], Duncan J. Wisniewski[1], George D. Spyropoulos[1§], Anastasios Polyravas[1], Jennifer N. Gelinas*[2,3], Dion Khodagholy*[1]

1. Department of Electrical Engineering, Columbia University, New York, NY 10027, USA
2. Department of Neurology, Columbia University Medical Center, New York, NY 10032, USA.
3. Institute for Genomic Medicine, Columbia University Medical Center, 630 W 168th St. New York, NY 10032, USA

† These authors contributed equally

§ Current address:

Department Information Technology, Waves, UGhent
Technology Campus, iGhent, Technologiepark 126, 9052 Zwijnaarde, Belgium

* Corresponding author:

Jennifer Gelinas: jng2146@cumc.columbia.edu
Dion Khodagholy: dk2955@columbia.edu

**Abstract**

Organic electronics can be biocompatible and conformable, enhancing the ability to interface with tissue. However, limitations of speed and integration have thus far necessitated reliance on silicon-based technologies for advanced processing, data transmission, and device powering. Here, we create a stand-alone, conformable, fully organic bioelectronic device capable of realizing these functions. This device is based on a novel transistor architecture that incorporates a vertical channel and miniaturized hydration access conduit to enable MHz signal range operation within densely packed integrated arrays in the absence of crosstalk (vertical internal ion-gated organic electrochemical transistor, vIGT). vIGTs demonstrated long-term stability in physiologic media, and were used to generate high performance integrated circuits. We leveraged the high speed and low voltage operation of vIGTs to develop alternating current-powered conformable circuity to acquire and wirelessly communicate signals. The resultant stand-alone device was implanted in freely moving rodents to acquire, process, and transmit neurophysiologic brain signals. Such fully organic devices have the potential to expand the utility and accessibility of bioelectronics to a wide range of clinical and societal applications.

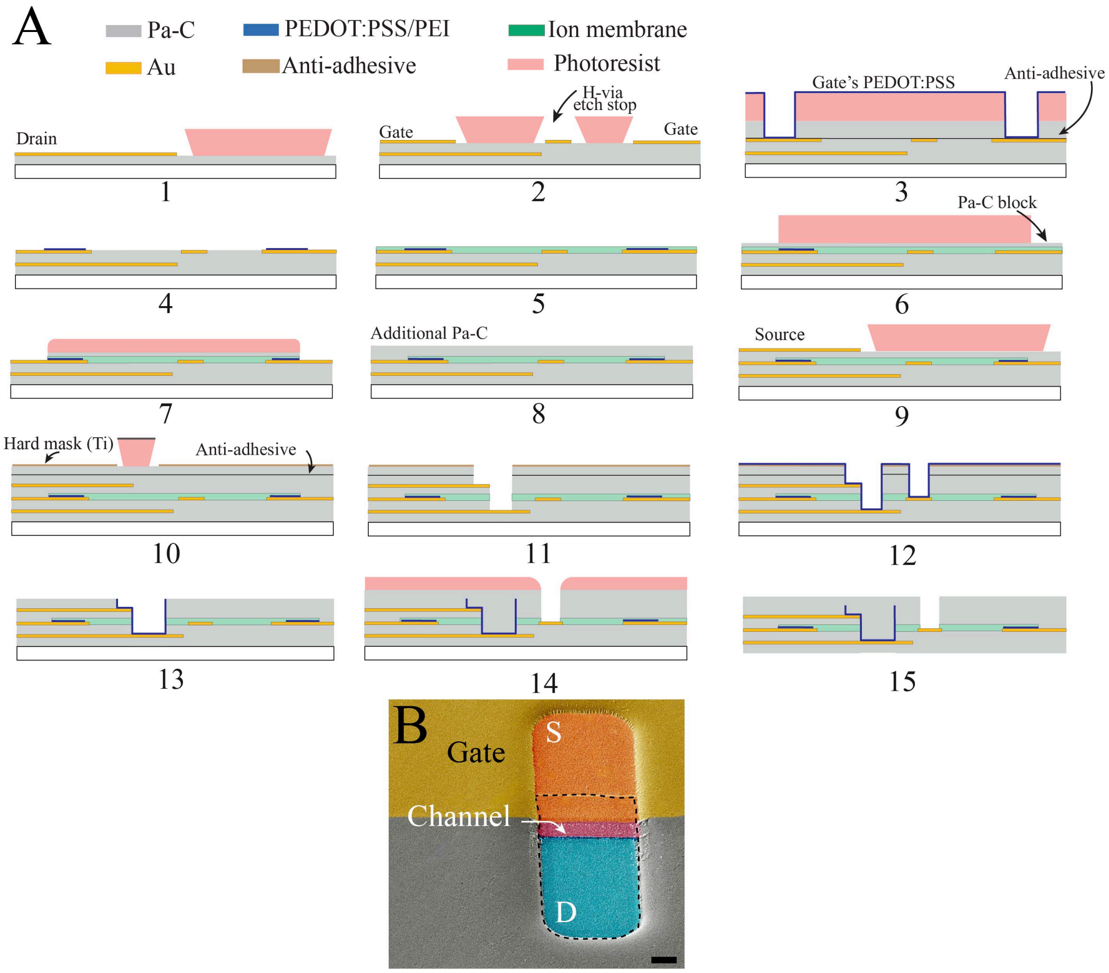


**Supplementary Figure 1: vIGT microfabrication process and SEM images.**
A) Optical lithography-based microfabrication process of sub-micrometer vIGTs.
1. Patterning of drain electrodes (Au/Ti;150/15 nm) via photolithography and lift-off process.
2. Deposition of Pa-C layer (300 nm) and patterning of the second metal layer serving as gate electrode, same parameters as 1.
3. Peel-off process for gate PEDOT:PSS patterning performed by lithography and $O_2$ plasma etching.
4. Finalized metal 2 layer with interconnects and gate electrodes coated with PEDOT:PSS.
5. Deposition of ion membrane (IM) layer by spin coating (purified chitosan: 250 nm).
6. Protection of IM by a thin Pa-C (<150 nm) layer and photolithographic pattering of the IM area.
7. $O_2$ plasma etching (180 W) of IM. A mild, 1-3 sccm $CH_4$/$SF_6$ mixture was used for removal of $SiO_2$ originating from the silane-based Pa-C adhesion promoter.
8. Addition of Pa-C layer to cover the unintended etch areas during IM plasma etch.
9. Patterning of source electrodes (Au/Ti;150/15 nm) via photolithography and lift-off process.
10. Deposition of Ti-based hard etch mask for defining the channel area on top of a peelable sacrificial Pa-C layer.
11. $O_2$ plasma etching (180 W) of IM (same as 5); Au source and drain contacts act as etch stops.
12. Deposition of the conducting polymer channel using spin coating.
13. Peel-off process of the sacrificial Pa-C layer made possible because of anti-adhesive treatment.
14. Photolithographic patterning and $O_2$ plasma etching of the H-via.
15) Final transistor architecture.
B) Top view SEM (30° tilt) of an individual vIGT channel during fabrication prior to PEDOT:PSS deposition (scale bar, 5 μm). The gate metal interlayer is represented by the yellow shaded region.

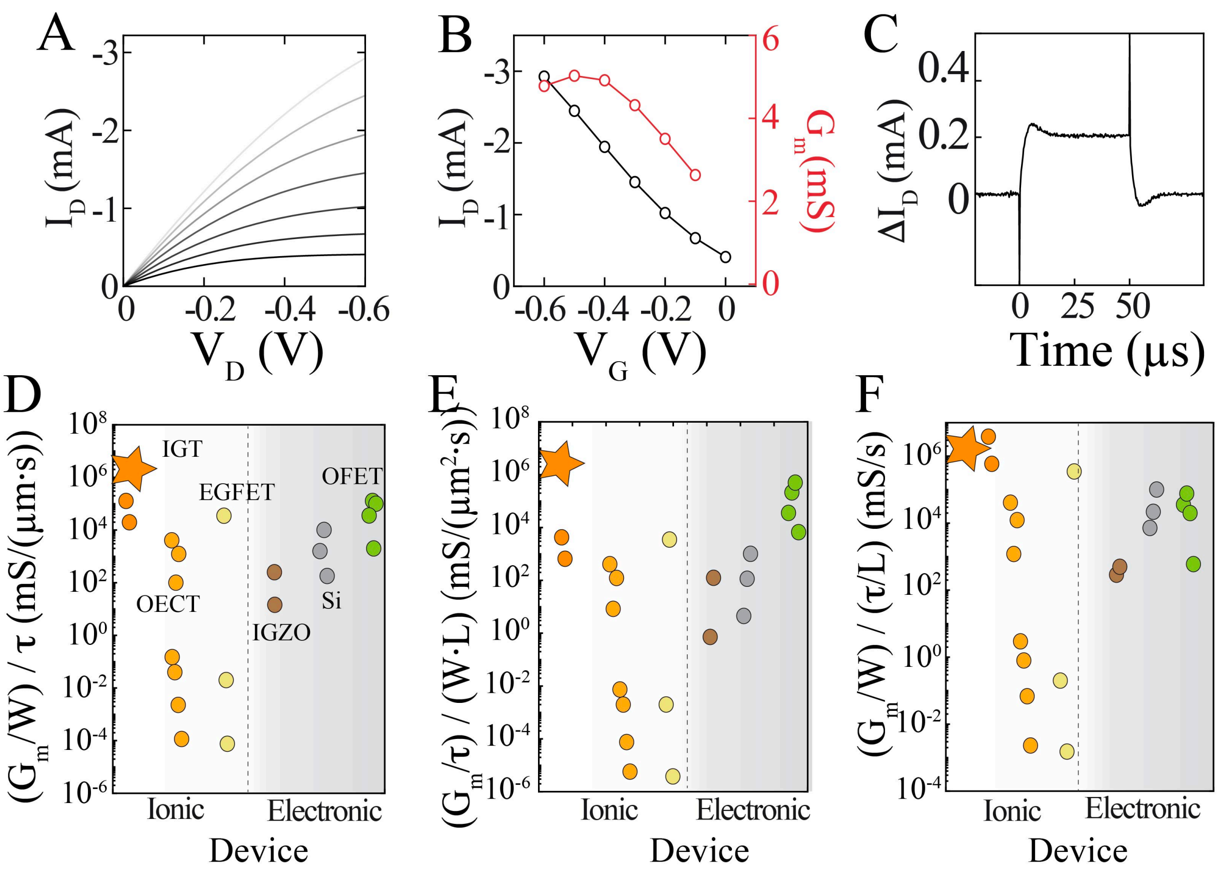


**Supplementary Figure 2: Output characteristics ($I_D$–$V_D$) and temporal response of an enhancement mode vIGT.**

A) Output characteristics ($I_D$-$V_D$) of an enhancement mode vIGT (L = 1 µm, W = 5 µm, thickness of PEDOT:PSS = 100 nm) for $V_G$ varying from 0 V (bottom curve) to -0.6 V (top curve) with a step of 0.1 V; color intensity corresponds to $V_G$ amplitude (left). Transfer curve for $V_D$ = -0.6 V (black) with corresponding transconductance (red), $G_m$ = 5.03 mS (right). Star represents vIGT.

B) Corresponding temporal response of the drain current ($I_D$) for $V_D$ = -0.6 V and $V_G$ pulse amplitude of 0.1 V.

C) Exponential fit of the vIGT drain current resulted in a time constant of 0.8 µs (right).

D) Expanded version of **Figure 1G.**

E) Performance of flexible transistors as characterized by the normalized ratio of transconductance and rise time vs. channel area. Same color scheme as **Figure 1G.**

F) Performance of flexible transistors as characterized by the ratio of the normalized transconductance by width and normalized rise time by channel length. Same color scheme as **Figure 1G.**

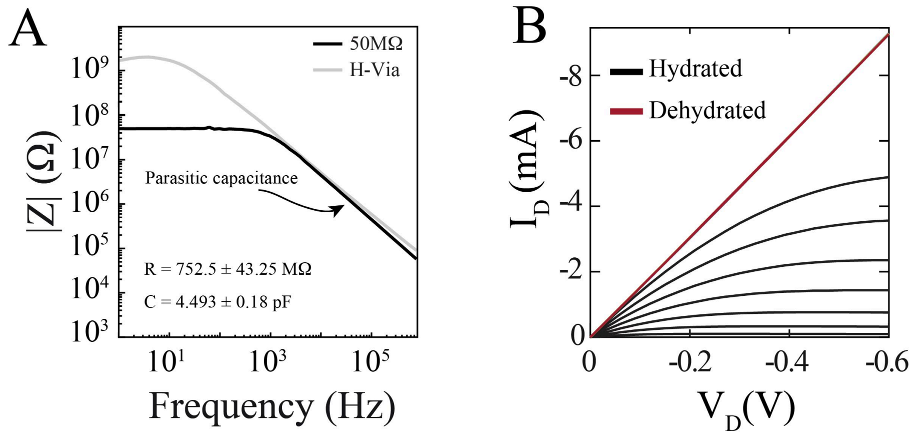


**Supplementary Figure 3: Electrochemical impedance characterization of vIGT and H-via.**

A) 4-electrode potentiostatic mode EIS on a ceramic-based 50 MΩ resistor (black). The EIS contains a capacitive component that is attributed to the instrument and setup parasitic capacitance. An RC model was used to extract this capacitance (~ 4.5 pF).
To measure the impedance of the H-via in a 4-electrode setting, we fabricated vIGTs without the conducting polymer channel connecting source and drain contacts. This set-up allowed utilization of source and drain contacts as 2 separate electrodes below the H-via, while a pair of Ag/AgCl and Pt wires served as the remaining electrodes above the H-via. The EIS measurement of the H-via was then compared to the pure resistor calibration measurements. The EIS fitting of the H-via curve had a capacitance value consistent with the parasitic capacitance extracted during the pure resistor measurements.

B) Comparison of output characteristics of a dehydrated channel (red) vs. a channel hydrated through the H-via (black).

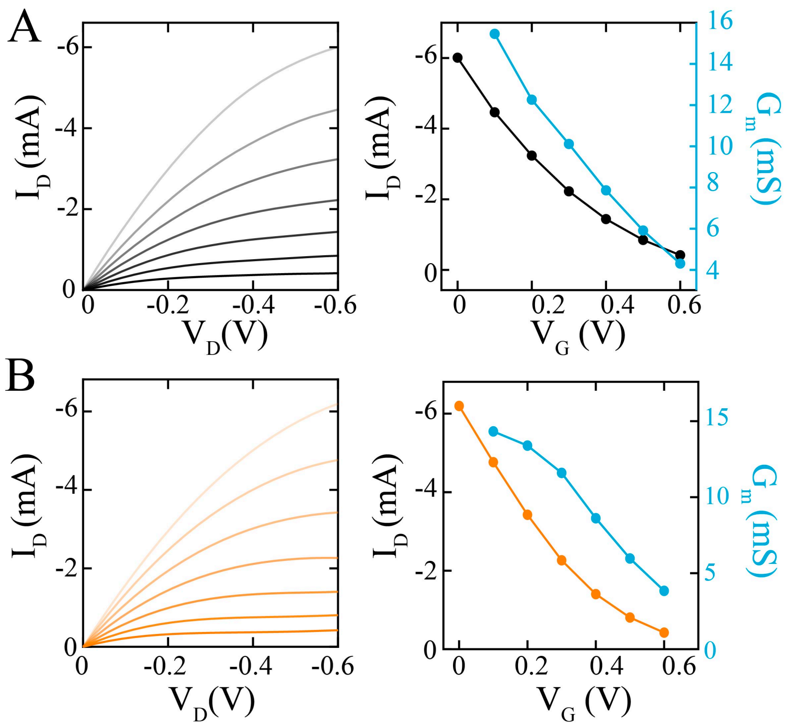


**Supplementary Figure 4: H-via does not impede vIGT electrical performance.**

A) Corresponding output characteristics ($I_D$-$V_D$) of the vIGT with channel fully exposed to electrolyte shown as the black curve in **Figure 2B**. $V_G$ varies from 0 V (top curve) to 0.6 V (bottom curve) with a step of 0.1 V; color intensity corresponds to $V_G$ amplitude (left). Transfer curve for $V_D = -0.6$ V (black) with corresponding transconductance (blue), $G_m^{max} = 15.45$ mS (right).

B) Corresponding output characteristics ($I_D$-$V_D$) of the vIGT with H-via exposure shown as the orange curve in **Figure 2B**. $V_G$ varies from 0 V (top curve) to 0.6 V (bottom curve) with a step of 0.1 V; color intensity corresponds to $V_G$ amplitude (left). Transfer curve for $V_D = -0.6$ V (orange) with corresponding transconductance (blue), $G_m^{max} = 14.33$ mS (right).

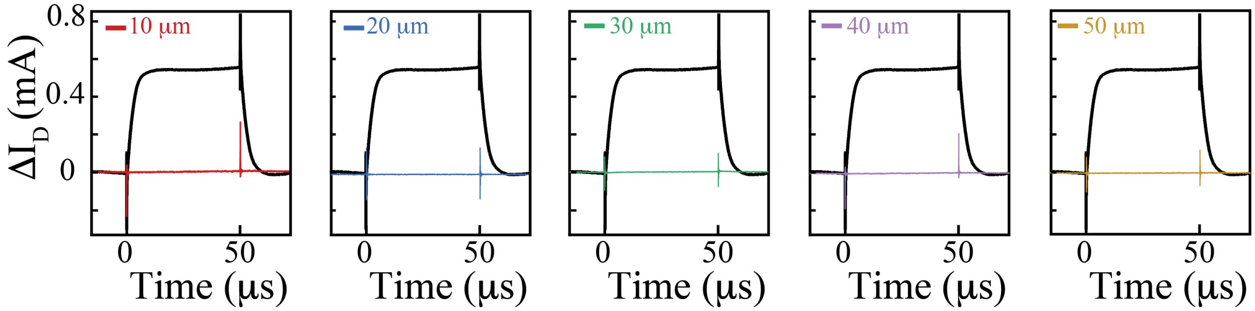


**Supplementary Figure 5: H-via enables creation of densely packed, independently-addressable vIGTs without inter-transistor crosstalk.**

Corresponding temporal responses of the drain current ($I_D$) of the vIGT shown in **Figure 2F** with input applied to its own gate (black curves) vs. input applied to neighboring transistor gates at channel-to-gate distances varying from 10 µm to 50 µm (colored curves). Transistor was operated at $V_D$ = – 0.4 V, with pulsed $V_G$ between 0 - 0.6 V (n = 128 pulses per current measurement). Transistors had identical geometry (W/L = 5/0.8 µm, d = 100 nm).

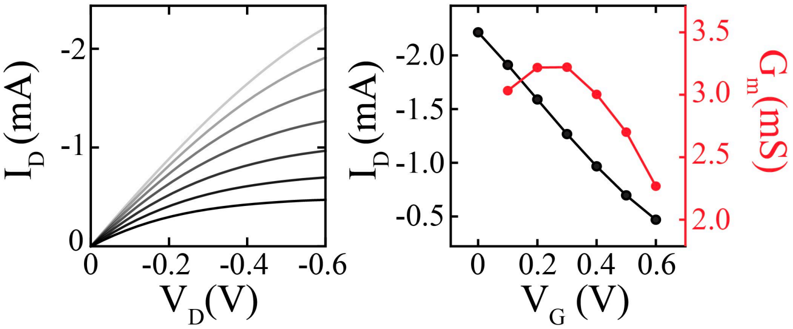


**Supplementary Figure 6: vIGT performance exhibits long-term stability in physiologic conditions.**

Output characteristics ($I_D$-$V_D$) of the vIGT shown in **Figure 1E** after 362 days in PBS solution. $V_G$ varies from 0 V (top curve) to 0.6 V (bottom curve) with a step of 0.1 V; color intensity corresponds to $V_G$ amplitude (left). Transfer curve for $V_D = -0.6$ V (black) with corresponding transconductance (red), $G_m^{max} = 3.22$ mS (right).

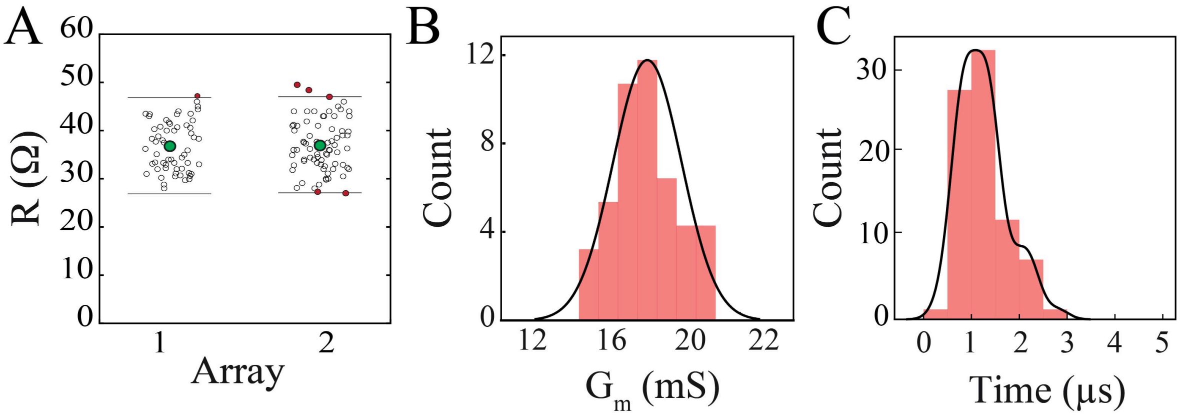


**Supplementary Figure 7: Wafer-scale fabrication of vIGTs with consistent performance.**

A) Distribution of the channel resistances of two independent arrays of transistors. The black lines demarcate the acceptable variation in resistance (1 × std). Green filled symbols mark the mean and the red circles highlight the rejected transistors (n = 138 total devices with 6 rejected devices).
B) Histogram of the transconductance values for vIGTs (W/L = 15/0.8 μm) across multiple wafers. Superimposed black line shows the corresponding fitted distribution.
C) Histogram of temporal response values for vIGTs across multiple wafers. Superimposed black line shows the corresponding fitted distribution.

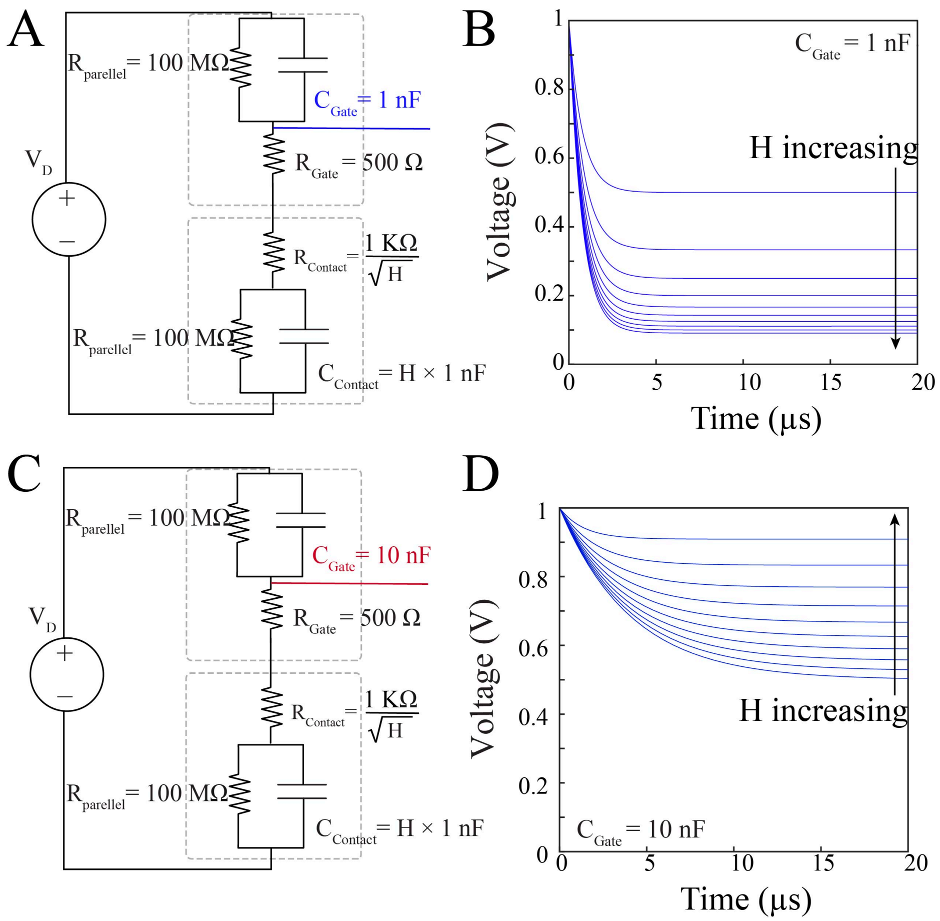


**Supplementary Figure 8: Modeling and equivalent circuit diagrams for gate and contact interactions.**

A) Circuit model of the gate and contact resistance, as well as capacitance, when the gate has a smaller area than the contact.
B) Simulation output used to measure the time constant as H (contact length) is increased from 1 to 10 in the gate < contact area condition.
C) Circuit model of the gate and contact resistance, as well as capacitance, when the gate has a larger area than the contact.
D) Simulation output used to measure the time constant as H (contact length) is increased from 1 to 10 in the gate > contact area condition.

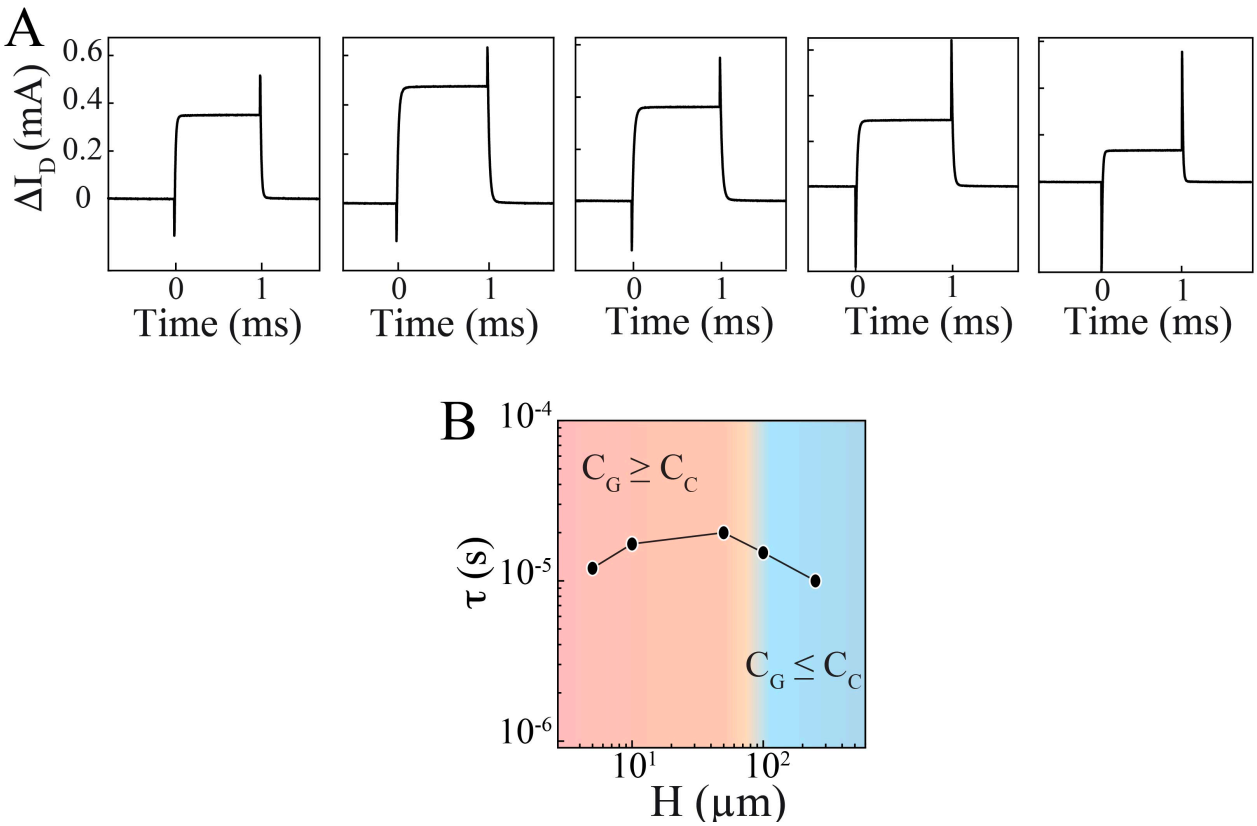


**Supplementary Figure 9: Contact area has a non-linear relationship with temporal response.**

A) Relationship between contact length (H) and temporal response for a horizonal channel IGT (W/L = 100 / 100 μm). Corresponding temporal response of the vIGTs with H = 5, 10, 50, 100, 250 μm.
B) Time constant of the horizontal IGT as a function of contact area (W/L = 100 / 100 μm).

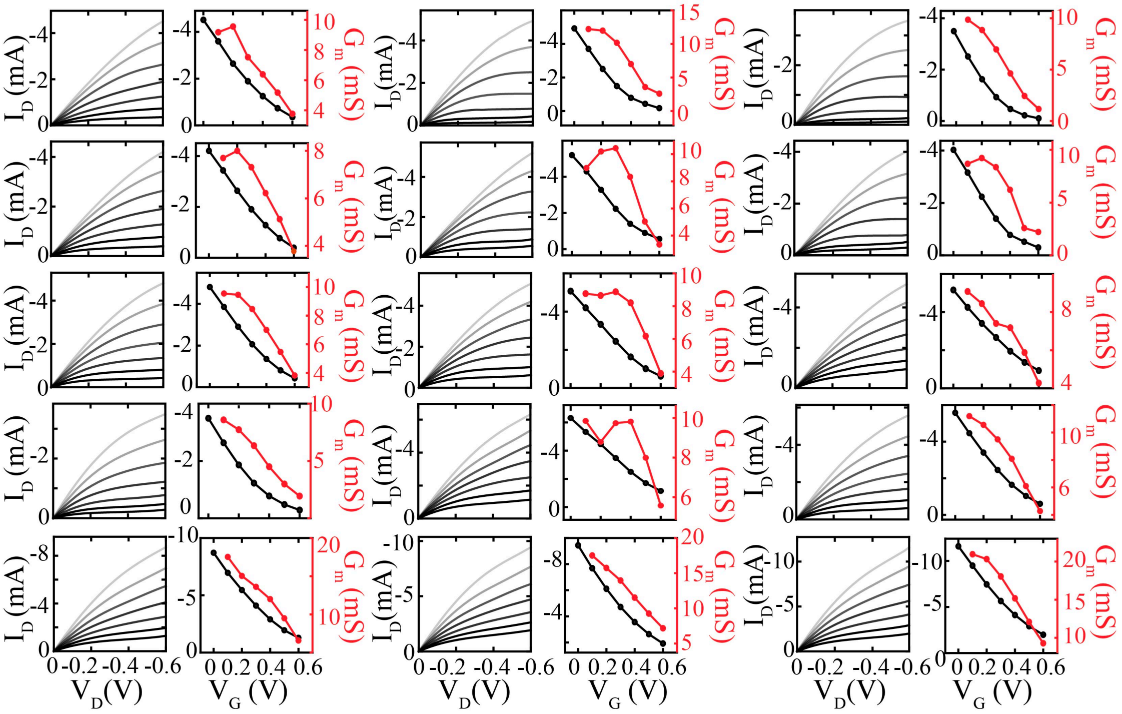


**Supplementary Figure 10: Effect of contact area on temporal response of vIGT.**

Corresponding output characteristics ($I_D$-$V_D$) of the vIGT shown in **Figure 3C**. Each row corresponds to five different contact length values (12 µm, 30 µm, 110 µm, 250 µm and 500 µm). Transistors in the same row have the same geometry for evaluation of consistency (n = 3).

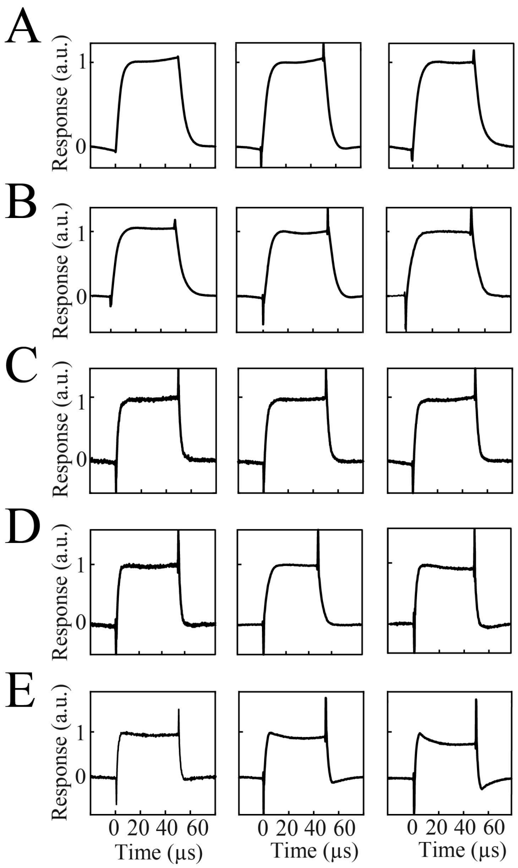


**Supplementary Figure 11: vIGTs with larger channel contact length (H) have faster temporal responses.**

A) Corresponding temporal responses of vIGTS with H = 12 μm shown in **Figure 3D,** τ = 3.7 μs ± 0.28 μs (n = 3, mean ± standard deviation).

B) Corresponding temporal responses of vIGTs with H = 30 μm shown in **Figure 3D,** τ = 3.0 μs ± 0.74 μs (n = 3, mean ± standard deviation).

C) Corresponding temporal responses of vIGTs with H = 110 μm shown in **Figure 3D,** τ = 1.7 μs ± 0.14 μs (n = 3, mean ± standard deviation).

D) Corresponding temporal responses of vIGTs with H = 250 μm shown in **Figure 3D,** τ = 1.3 μs ± 0.06 μs (n = 3, mean ± standard deviation).

E) Corresponding temporal responses of vIGTs with H = 500 μm shown in **Figure 3D,** τ = 0.8 μs ± 0.20 μs (n = 3, mean ± standard deviation).

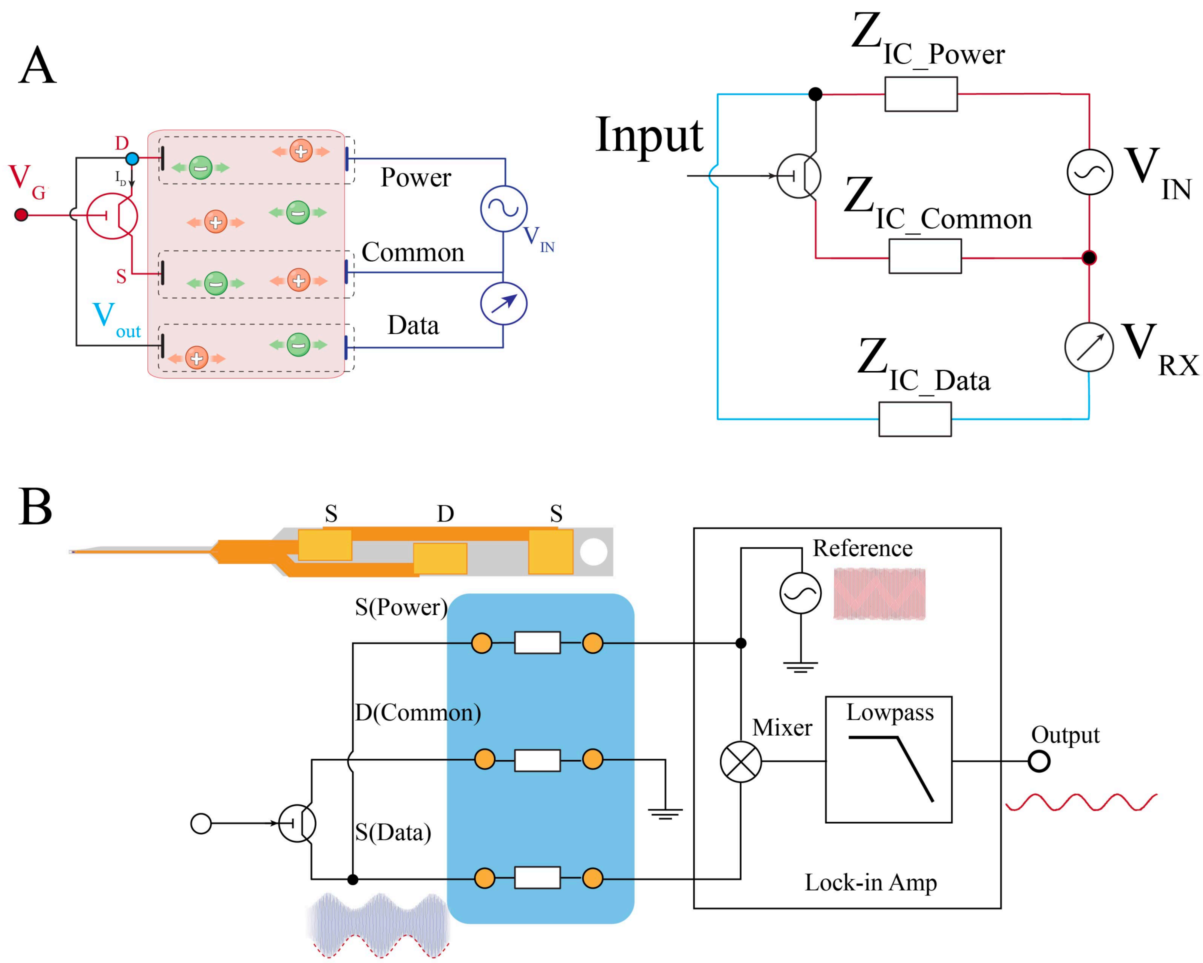


**Supplementary Figure 12: Equivalent circuit diagram of stand-alone vIGT.**

A) Simplified circuit model of the wireless vIGT-based stand-alone device. $Z_{IC\_Common}$ denotes impedance of grounding IC channel (source electrode). $Z_{IC\_Power}$ denotes impedance of primary IC channel for power delivery (drain electrode). $Z_{IC\_Data}$ denotes impedance of secondary IC channel for signal output.

B) Top view schematic of the vIGT-based wireless probe and the block diagram of the receiver.

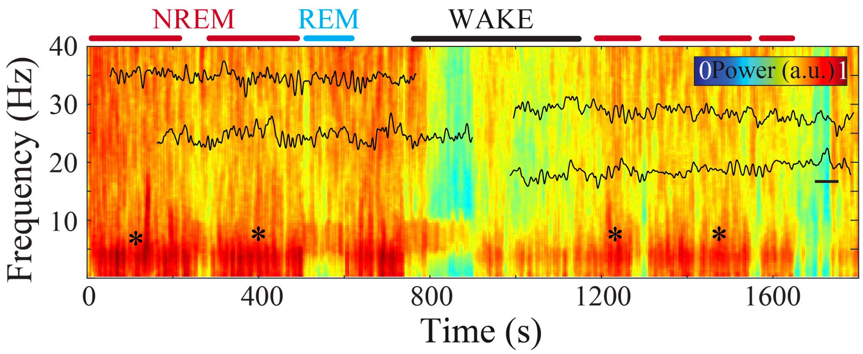


**Supplementary Figure 13: Neurophysiological data acquired and transmitted by stand-alone vIGT device *in vivo*.**

Continuous time-frequency spectrogram of neural data acquired and wirelessly transmitted using vIGT-based stand-alone device demonstrates characteristic LFP patterns corresponding to wakefulness, REM sleep, and NREM sleep. Superimposed raw time trace highlights spindle oscillations during NREM sleep (scale bar, 200 ms).

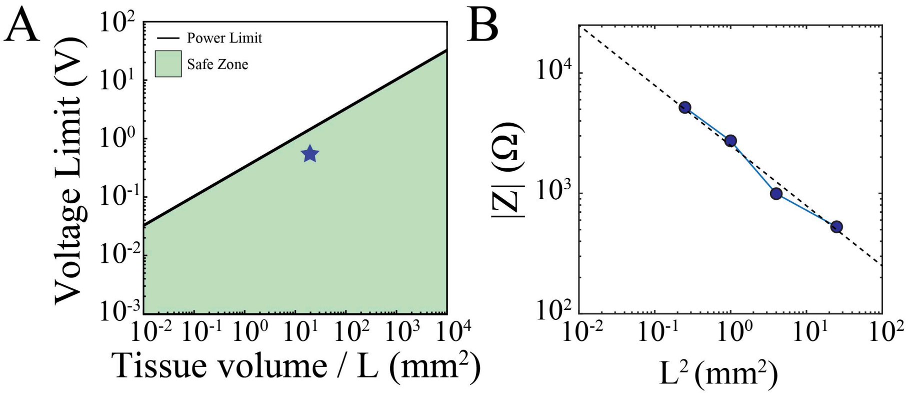


**Supplementary Figure 14: Stand-alone vIGT-based wireless implant complies with safety values for tissue specific energy absorption rate (SAR) as mandated by the International Commission on Non-Ionizing Radiation Protection (ICNIRP).**

A) Operating voltage limit of the stand-alone vIGT-based wireless implant as a function of the tissue volume between its electrodes and the size of the electrode (L). The black line indicates the safety boundary for the induced electric field in the tissue. The green shaded area highlights the compliant voltages for a given configuration. The star indicates the parameters used in our *in vivo* studies ($V_{TX}$ = 500 mV, L = 1 mm).

B) Measured (blue circles) and fitted (dashed line) impedance values as a function of electrode area ($L^2$) used for estimation of operating voltages.

| Device | $\tau$ (s) | $G_m^{max}$ (mS) | $(G_m^{max}/W)/\tau$ (mS/µm s) | L (µm) | W (µm) |
|---|---|---|---|---|---|
| V-IGT | $9.20\times10^{-7}$ | $3.87\times10^{0}$ | $2.10\times10^{6}$ | $8.00\times10^{-1}$ | $2.00\times10^{0}$ |
| E-IGT[16] | $8.60\times10^{-6}$ | $5.49\times10^{0}$ | $1.28\times10^{5}$ | $3.00\times10^{1}$ | $5.00\times10^{0}$ |
| DIGT[15] | $2.80\times10^{-5}$ | $6.62\times10^{0}$ | $1.97\times10^{4}$ | $3.00\times10^{1}$ | $1.20\times10^{1}$ |
| DOECT[39,51] | $3.90\times10^{-5}$ | $1.60\times10^{0}$ | $4.10\times10^{3}$ | $1.00\times10^{1}$ | $1.00\times10^{1}$ |
| E-OECT[16] | $2.29\times10^{-4}$ | $5.74\times10^{0}$ | $1.00\times10^{2}$ | $1.20\times10^{1}$ | $2.50\times10^{2}$ |
| g2T-T[52] | $6.40\times10^{-4}$ | $7.90\times10^{0}$ | $1.23\times10^{3}$ | $1.00\times10^{1}$ | $1.00\times10^{1}$ |
| PTEBS:PEDOTS | $7.10\times10^{-2}$ | $1.19\times10^{1}$ | $1.49\times10^{-2}$ | $2.00\times10^{1}$ | $1.125\times10^{3}$ |
| S-aPEDOT[53] | $3.60\times10^{-1}$ | $1.62\times10^{1}$ | $4.00\times10^{-2}$ | $2.00\times10^{1}$ | $1.125\times10^{3}$ |
| $ZnO_2$[36] | 5.00E-07 | $3.5\times10^{0}$ | $3.50\times10^{4}$ | $10\times10^{0}$ | $200\times10^{0}$ |
| $ZnO_2$[54] | $5.00\times10^{-5}$ | $2.00\times10^{-4}$ | $2.00\times10^{-2}$ | $1.00\times10^{1}$ | $2.00\times10^{2}$ |
| BBL:PEI[55] | $1.67\times10^{-1}$ | $3.80\times10^{-1}$ | $2.28\times10^{-3}$ | $3.00\times10^{1}$ | $1.00\times10^{3}$ |
| PTEBS[53] | $6.90\times10^{0}$ | $9.00\times10^{-1}$ | $1.16\times10^{-4}$ | $2.00\times10^{1}$ | $1.125\times10^{3}$ |
| P3HT[56] | $1.00\times10^{-3}$ | $1.52\times10^{-5}$ | $7.60\times10^{-5}$ | $2.00\times10^{1}$ | $2.00\times10^{2}$ |
| Si NM[57] | $1.50\times10^{-6}$ | $5.00\times10^{-1}$ | $1.01\times10^{4}$ | $1.00\times10^{1}$ | $3.30\times10^{1}$ |
| V-OFET[58] | $1.00\times10^{-7}$ | $1.00\times10^{-3}$ | $2.00\times10^{3}$ | $3.00\times10^{-1}$ | $5.00\times10^{-0}$ |
| OEFT[59] | $5.6\times10^{-7}$ | $8.00\times10^{-3}$ | $1.78\times10^{2}$ | $1.20\times10^{-1}$ | $80\times10^{0}$ |
| OEFT[60] | $5.60\times10^{-8}$ | $4.00\times10^{-1}$ | $3.57\times10^{4}$ | $1.00\times10^{0}$ | $200\times10^{0}$ |
| OEFT[60] | $4.70\times10^{-8}$ | $6.00\times10^{-1}$ | $1.28\times10^{5}$ | $6.00\times10^{-1}$ | $100\times10^{0}$ |
| OEFT[61] | $4.00\times10^{-8}$ | $2.00\times10^{-1}$ | $1.00\times10^{5}$ | $2.00\times10^{-1}$ | $50\times10^{0}$ |
| Si NM[62] | $5.00\times10^{-6}$ | $1.80\times10^{-1}$ | $1.80\times10^{2}$ | $4.00\times10^{1}$ | $2.00\times10^{2}$ |
| Si NM[63] | $2.60\times10^{-6}$ | $3.30\times10^{-1}$ | $1.59\times10^{3}$ | $1.38\times10^{1}$ | $8.00\times10^{1}$ |
| IGZO[64] | $6.92\times10^{-8}$ | $1.00\times10^{-4}$ | $1.45\times10^{1}$ | $2.00\times10^{1}$ | $1.00\times10^{2}$ |
| IGZO[65] | $1.00\times10^{-5}$ | $3.00\times10^{0}$ | $2.50\times10^{2}$ | $2.00\times10^{0}$ | $1.2\times10^{3}$ |

**Supplementary Table 1: Electrical and geometrical characteristics of flexible transistors shown in Figure 1E.**

| Material | Density (transistors/cm$^2$) |
|---|---|
| Si NMOS[66] | $2.424\times10^3$ |
| Si NM[57] | $9.090\times10^3$ |
| EGOFET ZnO[54] | $2.0\times10^2$ |
| IGZO TFTs[67] | $9.5169\times10^4$ |
| C-OTFT[68] | $6.0\times10^1$ |
| OECT[69] | $1.0\times10^2$ |
| OECT[70] | $3.25\times10^2$ |
| CNT FET[71] | $4.0\times10^3$ |
| OTFT[72] | $6.25\times10^3$ |
| gSGFET[73–75] | $1.111\times10^3$ |
| OFET[76] | $4.2\times10^4$ |
| VIGT | $1.55586\times10^5$ |
| OECT[77] | $3.1746\times10^4$ |
| OECT-OFET[78] | 25 |

**Supplementary Table 2: Density of flexible transistors shown in Figure 4C.**

51. Khodagholy, D. *et al.* In vivo recordings of brain activity using organic transistors. *Nat Commun* **4**, 1575 (2013).
52. Nielsen, C. B. *et al.* Molecular Design of Semiconducting Polymers for High-Performance Organic Electrochemical Transistors. *J Am Chem Soc* **138**, 10252–10259 (2016).
53. Zeglio, E. *et al.* Conjugated Polyelectrolyte Blends for Electrochromic and Electrochemical Transistor Devices. *Chemistry of Materials* **27**, 6385–6393 (2015).
54. Zare Bidoky, F. *et al.* Sub-3 V ZnO Electrolyte-Gated Transistors and Circuits with Screen-Printed and Photo-Crosslinked Ion Gel Gate Dielectrics: New Routes to Improved Performance. *Adv Funct Mater* **30**, (2020).
55. Yang, C. Y. *et al.* A high-conductivity n-type polymeric ink for printed electronics. *Nat Commun* **12**, (2021).
56. Cho, J. H. *et al.* High-capacitance ion gel gate dielectrics with faster polarization response times for organic thin film transistors. *Advanced Materials* **20**, 686–690 (2008).
57. Song, E. *et al.* Flexible electronic/optoelectronic microsystems with scalable designs for chronic biointegration. *Proc Natl Acad Sci U S A* **116**, 15398–15406 (2019).
58. Kleemann, H., Schwartz, G., Zott, S., Baumann, M. & Furno, M. Megahertz operation of vertical organic transistors for ultra-high resolution active-matrix display. *Flexible and Printed Electronics* **5**, (2020).
59. Haldar, T. *et al.* High-gain, low-voltage unipolar logic circuits based on nanoscale flexible organic thin-film transistors with small signal delays. *Sci Adv* **9**, (2023).
60. Borchert, J. W. *et al.* Flexible low-voltage high-frequency organic thin-film transistors. *Sci Adv* **6**, (2020).
61. Zschieschang, U., Waizmann, U., Weis, J., Borchert, J. W. & Klauk, H. Nanoscale flexible organic thin-film transistors. *Sci. Adv* **8**, 9845 (2022).
62. Viventi, J. *et al.* Flexible, foldable, actively multiplexed, high-density electrode array for mapping brain activity in vivo. *Nat Neurosci* **14**, 1599–1605 (2011).
63. Fang, H. *et al.* Capacitively coupled arrays of multiplexed flexible silicon transistors for long-term cardiac electrophysiology. *Nat Biomed Eng* **1**, 38 (2017).
64. Suresh, A. *et al.* Fast all-transparent integrated circuits based on indium gallium zinc oxide thin-film transistors. *IEEE Electron Device Letters* **31**, 317–319 (2010).
65. Rahaman, A., Chen, Y., Hasan, M. M. & Jang, J. A High Performance Operational Amplifier Using Coplanar Dual Gate a-IGZO TFTs. *IEEE Journal of the Electron Devices Society* **7**, 655–661 (2019).
66. Chiang, C. H. *et al.* Development of a neural interface for high-definition, long-term recording in rodents and nonhuman primates. *Sci Transl Med* **12**, 1–13 (2020).
67. Biggs, J. *et al.* A natively flexible 32-bit Arm microprocessor. *Nature* **595**, 532–536 (2021).
68. Kwon, J. *et al.* Three-dimensional monolithic integration in flexible printed organic transistors. *Nat Commun* **10**, 1–10 (2019).
69. Andersson Ersman, P. *et al.* All-printed large-scale integrated circuits based on organic electrochemical transistors. *Nat Commun* **10**, 1–9 (2019).
70. Lee, W. *et al.* Transparent, conformable, active multielectrode array using organic electrochemical transistors. *Proc Natl Acad Sci U S A* **114**, 10554–10559 (2017).
71. Liu, L. *et al.* Aligned, high-density semiconducting carbon nanotube arrays for high-performance electronics. *Science* **368**, 850–856 (2020).

72. Chen, G. *et al.* Micron-Scale Resolution Image Sensor Based on Flexible Organic Thin Film Transistor Arrays via Femtosecond Laser Processing. *IEEE Electron Device Letters* **43**, 248–251 (2022).
73. Schaefer, N. *et al.* Improved metal-graphene contacts for low-noise, high-density microtransistor arrays for neural sensing. *Carbon N Y* **161**, 647–655 (2020).
74. Bonaccini Calia, A. *et al.* Full-bandwidth electrophysiology of seizures and epileptiform activity enabled by flexible graphene microtransistor depth neural probes. *Nature Nanotechnology 2021 17:3* **17**, 301–309 (2021).
75. Kostarelos, K., Vincent, M., Hebert, C. & Garrido, J. A. Graphene in the Design and Engineering of Next-Generation Neural Interfaces. *Advanced Materials* **29**, (2017).
76. Zheng, Y. Q. *et al.* Monolithic optical microlithography of high-density elastic circuits. *Science* **373**, 88–94 (2021).
77. Khodagholy, D. *et al.* High speed and high density organic electrochemical transistor arrays. *Appl Phys Lett* **99**, 99–102 (2011).
78. Lee, W. *et al.* Integration of Organic Electrochemical and Field-Effect Transistors for Ultraflexible, High Temporal Resolution Electrophysiology Arrays. *Advanced Materials* **28**, 9722–9728 (2016).
79. [Data] Integrated internal ion-gated organic electrochemical transistors for stand-alone conformable bioelectronics, doi:10.17632/5yjgb8pt4r.1
